# American College of Cardiology and American Heart Association blood pressure categories—a systematic review of the relationship with adverse pregnancy outcomes in the first half of pregnancy

**DOI:** 10.1016/j.ajog.2022.10.004

**Published:** 2023-04

**Authors:** Laura J. Slade, Hiten D. Mistry, Jeffrey N. Bone, Milly Wilson, Maya Blackman, Nuhaat Syeda, Peter von Dadelszen, Laura A. Magee

**Affiliations:** a Department of Obstetrics and Gynaecology, Women’s and Children’s Hospital, Adelaide, Australia; b Department of Women and Children’s Health, School of Life Course and Population Health Sciences, Faculty of Medicine, King’s College London, London, United Kingdom; c British Columbia Children's Hospital Research Institute, The University of British Columbia, Vancouver, Canada; d Department of Obstetrics and Gynecology, The University of British Columbia, Vancouver, Canada

**Keywords:** cardiovascular systematic review, chronic hypertension in pregnancy, preeclampsia, pregnancy complications, pregnancy-induced hypertension

## Abstract

**Objective:**

A relationship between the 2017 American College of Cardiology and American Heart Association blood pressure thresholds and adverse pregnancy outcomes has been reported, but few studies have explored the diagnostic test properties of these cutoffs.

**Data Sources:**

We systematically searched electronic databases (from 2017 to 2021) for reports of blood pressure measurements in pregnancy, classified according to 2017 American College of Cardiology and American Heart Association criteria, and their relationship with pregnancy outcomes.

**Study Eligibility Criteria:**

Studies recording blood pressure at <20 weeks gestation were included.

**Methods:**

Meta-analyses were used to investigate the strength of the association between blood pressure cutoffs and adverse outcomes, and the diagnostic test properties were calculated.

**Results:**

Of 23 studies included, there was a stepwise relationship between the American College of Cardiology and American Heart Association blood pressure category (when compared with normal blood pressure of <120/80 mmHg) and the strength of the association with preeclampsia. The category of elevated blood pressure had a risk ratio of 2.0 (95% prediction interval, 0.8–4.8), the stage 1 hypertension category had a risk ratio of 3.0 (95% prediction interval, 1.1–8.5), and the stage 2 hypertension category had a risk ratio of 7.9 (95% prediction interval, 1.8–35.1). Between-study variability was related to the magnitude of the association with stronger relationships in larger studies at low risk of bias and with unselected populations with multiple routine blood pressure measurements. None of the systolic blood pressure measurements of <120 mmHg, <130/80 mmHg, or <140/90 mmHg were useful to rule out the development of preeclampsia (all negative likelihood ratios >0.2). Only a blood pressure measurement of ≥140/90 mmHg was a good predictor for the development of preeclampsia (positive likelihood ratio, 5.95). The findings were similar for other outcomes.

**Conclusion:**

Although a blood pressure of 120 to 140 over 80 to 90 mmHg at <20 weeks gestation is associated with a heightened risk for preeclampsia and adverse pregnancy outcomes and may assist in risk prediction in multivariable modelling, lowering the diagnostic threshold for chronic hypertension would not assist clinicians in identifying women at heightened risk.

## Introduction

Higher blood pressure (BP) measurements are associated with more adverse outcomes outside of and during pregnancy.^1^, ^2^, ^3^, ^4^ To stratify risk management and justify additional investigations and interventions, a systolic BP (sBP) ≥140 mmHg and diastolic BP (dBP) ≥90 mmHg have long been categorized as hypertension. In pregnancy, hypertension has been regarded as chronic if hypertension is recorded at <20 weeks gestation and as gestational if hypertension first appears ≥20 weeks gestation. When gestational hypertension occurs with proteinuria, another maternal end-organ complication, or uteroplacental dysfunction, the criteria are met for classification as preeclampsia, the hypertensive disorder of pregnancy associated with the highest maternal and perinatal risks.^5^

**AJOG at a Glance**

**Why was this study conducted?**

A relationship between the 2017 American College of Cardiology and the American Heart Association blood pressure (BP) thresholds and adverse pregnancy outcomes has been reported, but few studies have explored the diagnostic test properties of these cutoffs.

**Key findings**

Although higher BP values <20 weeks gestation is associated with adverse outcomes, lowering the threshold for chronic hypertension would not improve the identification of women and babies at risk. No abnormal BP threshold <20 weeks gestation, including the current 140/90 mmHg threshold, provides reassurance about the development of preeclampsia or adverse pregnancy outcomes.

**What does this add to what is known?**

Our findings do not support lowering the BP level component of the definition for chronic hypertension in pregnancy to below the current 140/90 mmHg threshold.

In 2017, the American College of Cardiology (ACC) and the American Heart Association (AHA) recommended lowering the BP thresholds for diagnosis of hypertension outside of pregnancy based on the association of these lower BP values with elevated cardiovascular risk. BP was considered to be normal if <120/80 mmHg, and the traditional definition of hypertension (sBP ≥140 mmHg and diastolic BP ≥90 mmHg) has been termed stage 2 hypertension. Two new BP categories were created, namely elevated BP (sBP, 120–129 mmHg and dBP <80 mmHg) and stage 1 hypertension (sBP, 130–139 mmHg and dBP, 80–89 mmHg).^6^

Since 2017, the diagnostic criteria for hypertension from a sBP ≥140 mmHg and a diastolic BP ≥90 mmHg have not been changed in any of the pregnancy hypertension guidelines.^7^ In 2019, the American College of Obstetricians and Gynecologists (ACOG) called for investigation into the consequences of adopting the 2017 ACC-AHA BP criteria. Many studies have been published, evaluating the association between preeclampsia (and other adverse pregnancy outcomes) and elevated BP, stage 1 hypertension, or a combination of both, particularly when BP was measured <20 weeks gestation.^8^, ^9^, ^10^, ^11^, ^12^, ^13^, ^14^

## Objective

We aimed to evaluate whether the definition of chronic hypertension in pregnancy should be revised according to the ACC-AHA criteria. Here, we present a systematic review of published studies on the association of BP values measured before 20 weeks gestation, classified according to ACC-AHA BP criteria, with preeclampsia and other adverse pregnancy outcomes.

## Materials and Methods

This systematic review was prospectively registered with the International Prospective Register of Systematic Reviews under identifier CRD42021229131.

### Eligibility criteria, information sources, search strategy

We searched Ovid MEDLINE, PubMed, Embase, Cumulative Index to Nursing and Allied Health Literature, Cochrane Central Register of Controlled Trials, Latin American and Caribbean Health Sciences Literature, and International Clinical Trials Registry Platform from January 1, 2017 to January 15, 2021, for all original research reports in peer-reviewed journals addressing BP in pregnancy according to the 2017 ACC/AHA guideline criteria.^6^ The search was updated monthly throughout the data collection and synthesis phase of the review up to September 9, 2021. The search terms used were (“American College of Cardiology/American Heart Association” OR “ACC/AHA” OR “Stage 1 hypertension” OR “Stage 2 hypertension” OR “prehypertension”) AND (“{Pregnancy [mh] OR Pregnan∗ OR Gestation∗ OR pregnant women[mh] OR Pregnancy Complications[mh] OR “Postpartum Period”[Mesh] OR Puerperium OR postpartum OR “Peripartum Period”[Mesh] OR Peripartum∗ OR Perinatal Care[mh] OR perinatal} AND {hypertension, hypertensive disorders of pregnancy, pregnancy-induced hypertension, preeclampsia, pregnancy toxemias, OR gestational hypertension}). The final list was confirmed using Google Scholar, using “pregnancy” AND (“prehypertension” OR “stage 1 hypertension” OR “ACC/AHA” OR “American College of Cardiology/American Heart Association”). There were no language restrictions. In addition, reference lists of included papers were searched for additional eligible publications.

### Study selection

Included were reports of BP measurements in pregnancy before 20 weeks gestation as defined by study authors, classified according to the 2017 ACC/AHA criteria, and their relationship with maternal or fetal and newborn outcomes in pregnancy.

BP was categorized as normal (sBP <120 mmHg and dBP <80 mmHg), elevated BP (sBP 120–129 mmHg and dBP <80 mmHg), stage 1 hypertension (sBP 130–139 mmHg and/or dBP 80–89 mmHg), or stage 2 hypertension (sBP ≥140 mmHg and/or dBP ≥90 mmHg), which was also subdivided into nonsevere (sBP 140–159 mmHg and/or dBP 90–109 mmHg) and severe (sBP ≥160 mmHg and/or dBP ≥110 mmHg).^2^ A diagnosis of stage 2 hypertension was confirmed by a prescription of antihypertensive therapy (because there is no precedent for routine treatment of BP <140/90 mmHg).

Excluded were case reports, duplicate publications (including the more complete or recent data set), and studies that presented BP as a continuous variable without categorization.

### Outcomes

The main outcomes were preeclampsia (as defined by individual authors), serious maternal complications (composite as defined by individual authors, including cerebrovascular accident, cortical blindness, retinal detachment, pulmonary edema, acute kidney injury, liver capsule hematoma or rupture), preterm birth, fetal and newborn death, small-for-gestational age (SGA) infants, and admission to a neonatal intensive care unit (NICU).

The secondary maternal outcomes were miscarriage; maternal death; eclampsia; hemolysis, elevated liver enzymes, and low platelet (HELLP) syndrome; admission to intensive care; intubation or ventilation (other than for childbirth); placental abruption; cesarean delivery; and postpartum hemorrhage.

The secondary neonatal outcomes were respiratory support and neonatal seizures.

All outcome definitions specified by the study authors were accepted and catalogued. We did not evaluate severe preeclampsia separately from preeclampsia because of the international move away from use of an outcome that is variably defined^15^; instead, we used the outcome only when information on all combined preeclampsia cases was unavailable.

Severe maternal morbidities (eg, acute kidney injury) were not included individually; although they are core maternal outcomes in pregnancy hypertension, these are reported infrequently and also occur infrequently.

### Data extraction

Search results were reviewed by 3 reviewers (L.S., M.B., and N.S.) and the full text of all relevant studies were retrieved and reviewed for inclusion. Any disagreement about the eligibility of studies was resolved through consensus with the remainder of the review team. Authors were contacted for clarification when required.

Data were abstracted by 2 reviewers for each included study using a review-specific form to collect characteristics of the study, patients enrolled (including type of hypertension), BP measurement details (and treatment, if applicable), and outcome definitions. Data abstraction was undertaken by all the authors with each study having data extracted by 2 authors. Any disagreement was addressed by consulting the remainder of the group to reach a consensus.

Study authors were contacted for information that was unclear or to enquire about accessing data for individual outcomes if only composite outcomes were reported.

### Assessment of risk of bias

Methodologic quality was assessed by 2 reviewers, independently, using the Quality Assessment of Diagnostic Accuracy Studies 2 (QUADAS-2) tool for primary diagnostic accuracy studies.^16^ Any disagreement was resolved by a third reviewer. Studies were classified as being at high risk of bias overall if determined to be at high risk in any of the domains, at low risk of bias if judged to be at low risk in all the domains, and as unclear risk of bias when the risk in any category was uncertain.^17^

### Data synthesis and meta-analysis

Our primary aim was to assess the usefulness of the ACC-AHA BP criteria for abnormal BP <20 weeks gestation by estimating the strength of the association with adverse outcomes and the associated diagnostic test properties. To undertake meaningful comparisons, we pooled outcome data when evaluated in more than 3 studies.

To evaluate the association between each BP category and adverse outcomes, we conducted a meta-analysis using the Mantel-Haenszel method for combing risk ratios.^18^ Results were summarized using pooled risk ratios (RRs) and 95% prediction intervals. Heterogeneity was summarized using the *I*^2^ statistic and classified as may not be important (*I*^2^ <40%), may represent moderate heterogeneity (30%–60%), may represent substantial heterogeneity (50%–90%), and considerable (≥75%).^19^

To evaluate the diagnostic test properties of the lower limit of each BP category as a cutoff (in the way that we currently treat a BP of 140/90 mmHg), we estimated each study’s sensitivity, specificity, and positive (+LR) and negative (−LR) likelihood ratios. Pooled estimates of the likelihood ratios were calculated from the estimated sensitivity and specificity from the bivariate random effects models. Confidence intervals were computed using 100,000 Markov Chain Monte Carlo resamples from the joint bivariate normal distribution of the sensitivity and specificity.^20^, ^21^, ^22^ All effect measures were estimated with 95% confidence intervals (CIs). Diagnostic tests properties were interpreted as excellent when +LR ≥10.0 (to rule in the outcome) or −LR <0.10 (to rule in the outcome), good when +LR ≥5.0 or −LR <0.20, and fair hen +LR was between 2.0 and 4.9 or −LR was between 0.20 and 0.50.^23^

Publication bias for each main outcome was assessed when there were at least 10 relevant studies that reported on the outcome. Funnel plots were used to assess asymmetry visually, and if asymmetry were suggested, we performed exploratory analyses to investigate it using Egger’s test and, if necessary, the trim and fill method.^24^

Moderation was evaluated descriptively in subgroup analyses that accounted for potential differences in the study characteristics (such as quality or origin), baseline characteristics of the participants (such as body mass index [BMI] or comorbidities), potential co-interventions (such as aspirin), or outcome definitions.

All analyses were conducted using the meta^25^ and mada^26^ packages in R statistical software (R Core Team, Vienna, Austria).^25^^,^^27^

## Results

### Study selection and characteristics

A total of 529 publications were identified in the literature search from January 1, 2017 to September 9, 2021, of which 57 duplicates were removed and 420 were excluded following abstract screening, because they were case reports or review articles (n=107), did not report the relevant outcomes (n=221), or had no appropriate comparison group (n=92). Of 52 articles selected for full-text review, further exclusions (including removal of duplicate publications^28^) left 23 studies that reported on the association between ACC-AHA BP categories based on BP measurements before 20 weeks gestation and adverse pregnancy outcomes.^8^, ^9^, ^10^, ^11^^,^^13^^,^^14^^,^^29^, ^30^, ^31^, ^32^, ^33^, ^34^, ^35^, ^36^, ^37^, ^38^, ^39^, ^40^, ^41^, ^42^, ^43^, ^44^, ^45^

### Study characteristics and risk of bias

As shown in Table 1, approximately half the studies were retrospective observational designs, conducted in single centers, and primarily in high-income countries, mostly from America (12/23). Just more than half the studies were externally funded. All were included as full publications with a few supplemented with unpublished data. The majority of studies were at high risk of bias, based primarily on their patient selection; for details of the assessments based on the 4 domains of QUADAS-2, refer to Table S1. Each included study was reasonably large, reporting on a median of almost 10,000 pregnancies and collectively reporting on more than 700,000 pregnancies. Most participants were enrolled in the second trimester with only a few recruited in the first trimester. About one-third of studies stated their intention to enroll a low-risk cohort; however, almost all excluded women with a multiple pregnancy, half of the studies excluded women with known chronic hypertension, and in up to a quarter, women were excluded for other medical comorbidities. The rigor of BP measurement varied between studies (Table S2) with 13 of 23 using BP values collected under varied study conditions and the remainder using routinely recorded BP values from the medical record. Only 6 of 23 studies used an average of BP measurements as recommended,^5^ and 4 of 23 studies used a combination of multiple BP values across visits. No studies used home-based BP measurements.

**Table 1:** Characteristics of the included studies and their participants

Characteristics	<20 weeks gestation (N=23 studies)
Studies	
Type	
Observational retrospective controlled	12 (52)
Observational prospective controlled	2 (9)
Secondary analysis of randomized controlled trial	9 (39)
Center status	
Single center	11 (48)
Multiple centers <5	2 (10)
Multiple centers ≥5	11 (48)
Low- and middle-income country	4 (17)
Funding reported	
None	2 (10)
Not reported	6 (29)
Externally funding reported	13 (62)
Full publication	23 (100)
Includes unpublished data	4 (17)
QUADAS-2 overall risk of bias	
High risk	15 (65)
Low risk	4 (17)
Unclear	4 (17)
Participants	
Total	734,377
Number per study	9869 (2720–19,368)
Gestational age at study enrolment^a^	
1st trimester	5 (22)
2nd trimester	18 (78)
3rd trimester	Not applicable
Inclusion criteria	
Intention to enroll a low-risk cohort	8 (35%)
Exclusion criteria	
Multiple pregnancy	19 (83)
Chronic hypertension	11 (48)
Diabetes mellitus	5 (22)
Chronic kidney disease	5 (22)
Systemic lupus erythematosus	3 (13)
Demographics	
Maternal age reported	20 (87)
Age (y), median of reported mean or median (IQR)	29.6 (26.1–30.5) (20 studies)
Number of studies with ≥50% women aged ≥35 y	2 (12) (9 studies)
Nulliparity reported	21 (91)
Nulliparity, median % (IQR)	43.2 (36.2–74.4)
Number of studies with ≥50% nulliparous participants	9 (39)
BMI (kg/m^2^) reported^b^	20 (87)
BMI (kg/m^2^) (median of mean or median (IQR))	25.1 (23.4–26.2) (15 studies)
Overweight,^c^ median % (IQR)	11.5 (9.5–26.3) (8 studies)
Ethnicity reported, median % (IQR)	19 (83)
Non-Hispanic White	36.3 (25.0–50.7) (11 studies)
Non-Hispanic Black	19 (10.6–44.0) (11 studies)
Hispanic	16.4 (1.5–37.5) (11 studies)
Asian^d^	73.4 (13.6–99.0) (14 studies)
Other (median % (IQR) women)	2.9 (1.7–8.4) (8 studies)
N studies without predominance of any one ethnicity	3/19 (16)
Aspirin use for preeclampsia prevention reported^e^	10 (43)
Aspirin use (median % (IQR) women)	12.7 (5.8–40.3) (10 studies)
BP measurements^f^	
Nature of BP measurements used to define ACC-AHA level	
One measurement	16 (70)
Multiple––at least 2 consecutive measurements	4 (17)
Average of multiple measurements	5 (22)
Normal BP compared with:	
Elevated BP	9 (39)
Stage 1 hypertension	9 (39)
Stage 2 hypertension	7 (30)
BP cutoffs examined as new thresholds for abnormal BP:	
sBP 120 mmHg (with dBP <80 mmHg)	8 (35)
sBP 130 mmHg or dBP 80 mmHg	13 (57)
sBP 140 mmHg or dBP 90 mmHg	13 (57)
Results presented adjusted for baseline characteristics	16 (70)

Data are presented as number (percentage) of pregnancies and median (interquartile range) unless otherwise stated).

*ACC-AHA*, American College of Cardiology-American Heart Association; *BMI*, body mass index; *BP*, blood pressure; *dBP*, diastolic BP; *IQR*, interquartile range.

*Slade. The diagnostic accuracy of American College of Cardiology-American Heart Association blood pressure categories for identification of women and babies at risk. Am J Obstet Gynecol 2023*.

a Not mutually exclusive

b Institute of Medicine BMI criteria were used to define overweight (BMI, 25.0–29.9 kg/m^2^) and obesity (BMI, ≥30 kg/m^2^), unless ethnicity-refined norms were specified in the publication.^11^^,^^34^ BMI was recorded as a continuous value in 15 of 22 studies and as a cutoff for overweight or obesity in 8 of 22 studies with only 2 of 22 reporting both continuous and cutoff values

c The definition of overweight was as defined by the study authors

d Studies from China and Japan recorded as >90% Asian population without specific descriptors of study populations, because of low level of ethnic variation in these populations

e Aspirin use was reported in studies that enrolled women at increased risk for preeclampsia

f BP was categorized as normal (sBP <120 mmHg and dBP <80 mmHg), elevated BP (sBP 120–129 mmHg and dBP <80 mmHg), stage 1 hypertension (sBP 130–139 mmHg and/or dBP 80–89 mmHg), and stage 2 hypertension (sBP ≥140 mmHg and/or dBP ≥90 mmHg), which was also subdivided into nonsevere (sBP 140–159 mmHg and/or dBP 90–109 mmHg) and severe cases (sBP ≥160 mmHg and/or dBP ≥110 mmHg)2. A diagnosis of stage 2 hypertension was accepted when based on prescription of antihypertensive therapy (because there is no precedent for routine treatment of BP <140/90 mmHg).

### Synthesis of results

On average, women were about 30 years of age and almost half were nulliparous (Table 1). Most studies reported participants’ BMI with a median of normal to overweight. Most studies did not report ethnicity directly, but if they did, less than half of women were White and approximately 20% were Black or Asian (based primarily on assumptions about where the studies were conducted) and approximately 10% were Hispanic. Data on aspirin use for preeclampsia prevention was reported by close to half of the studies with a median use of 13% of participants.

About two-thirds of studies categorized BP based on 1 measurement (Table 1). Although different studies compared different combinations of ACC-AHA BP categories against each other (Table S3), normal BP was compared with either elevated BP (n=9 studies, 159,573 women), stage 1 hypertension (n=9 studies, 159,573 women), or stage 2 hypertension (n=7 studies, 92,620 women). Only the Community-Level Interventions in Preeclampsia (CLIP) trials subdivided stage 2 hypertension into severe (≥160/110 mmHg) and nonsevere cases (140–159/90–109 mmHg) as reported previously.^29^^,^^39^^,^^41^ New BP cutoffs were examined at sBP ≥120 mmHg (regardless of dBP; n=8 studies, 159,573 women), sBP ≥130 mmHg or dBP ≥80 mmHg (n=13 studies, 696,533 women), and sBP ≥140 mmHg or dBP ≥90 mmHg (n=13 studies, 176,348 women).

Overall, 16 studies reported adjustment for maternal covariates, most commonly maternal age, prepregnancy BMI, parity, and/or race and ethnicity^8^^,^^9^^,^^10^^,^^11^^,^^14^^,^^30^^,^^31^^,^^33^^,^^35^, ^36^, ^37^, ^38^^,^^42^, ^43^, ^44^^,^^46^; 7 of these studies reported both adjusted and unadjusted analyses.^8^^,^^9^^,^^31^^,^^42^^,^^43^^,^^46^^,^^47^ In all analyses, adjustment for maternal confounders reduced the association between BP categories and adverse outcomes, most significantly for stage 2 hypertension when reported.

### Preeclampsia

Preeclampsia was reported in 21 of 23 (91.0%) studies (661,828 women) at a median event rate of 4.0% (interquartile range [IQR], 2.4–6.3). Of these, 13 studies reported on all BP category comparisons (546,439 women). Most studies used the 2013 or 2019 ACOG definitions of preeclampsia, which did not require the presence of proteinuria for the diagnosis.

The Figure shows that when compared with normal BP, the risk for preeclampsia increased progressively with more advanced BP categories, based on BP measurements before 20 weeks gestation, from an RR of 1.9 (95% CI, 1.6–2.4; *I*^2^=87%) for elevated BP to an RR of 3.1 (95% CI, 2.4–3.9; *I*^2^=89%) for stage 1 hypertension and an RR of 8.0 for stage 2 hypertension (95% CI, 5.9–11.0; *I*^2^=91%). Although there was substantial between-study variation in the effects, it was not the presence of an association that differed between studies but rather the magnitude of the association that differed by more than could be expected by chance alone. A stronger relationship with preeclampsia was seen among studies at low risk of bias, those that were larger, and those with unselected populations for whom multiple routine (clinical) BP measurements were evaluated (Table S2).

**Figure:**
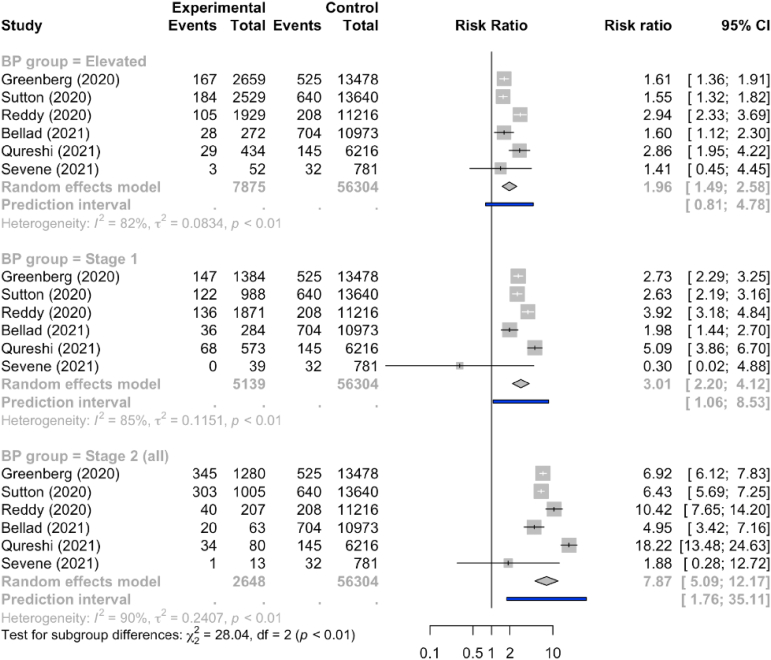
Relative risk for preeclampsia according to the ACC-AHA BP categories before 20 weeks gestation *ACC-AHA*, American College of Cardiology-American Heart Association; *BP*, blood pressure; *CI*, confidence interval. *Slade. The diagnostic accuracy of American College of Cardiology-American Heart Association blood pressure categories for identification of women and babies at risk. Am J Obstet Gynecol 2023*.

Table 2 shows that each cutoff with a sBP of 120 mmHg (with dBP <80 mmHg), an sBP 130 mmHg and/or a dBP of 80 mmHg, and an sBP of 140 mmHg and/or a dBP of 90 mmHg was associated with an increased risk for preeclampsia when compared with BP values below each of those cutoffs. However, neither an sBP <120/80 mmHg, a BP <130/80 mmHg, nor a BP <140/90 mmHg was useful to rule out development of preeclampsia, with −LR values all well above 0.2. In addition, neither an sBP ≥120 mmHg nor a BP ≥130/80 mmHg was useful to rule in development of preeclampsia; only a BP ≥140/90 mmHg was a good rule-in test for development of preeclampsia based on a pooled +LR of 5.95.

**Table 2:** Pooled relative risk and mean likelihood ratios for preeclampsia according to BP cutoffs at <20 weeks gestation based on ACC-AHA BP categories

Study	Ref	RR (95% CI); *I*^2^ (95% PI)	−LR (95% CI)	+LR (95% CI)
Diagnostic threshold: sBP 120 mmHg (and dBP <80 mmHg)				
Pooled	Normal (<120/80 mmHg)	3.1 (2.6–4.0); *I*^2^=10% (1.1–8.4)	0.69 (0.54–0.82)	2.42 (2.07–2.82)
Bellad et al,^29^ 2020		2.1 (1.7–2.6)	0.94 (0.92–0.96)	2.2 (1.73–2.68)
Gonzalez-Valencia et al,^32^ 2020		4.1 (1.9–8.9)	0.55 (0.35–0.89)	2.63 (1.72–4.02)
Greenberg et al,^9^ 2021		3.2 (2.8–3.5)	0.60 (0.57–0.64)	2.10 (1.99–2.22)
Qureshi et al,^39^ 2020		5.2 (4.1–6.5)	0.61 (0.54–0.68)	3.49 (3.04–4.00)
Reddy et al,^13^ 2020		3.8 (3.2–4.5)	0.57 (0.51–0.63)	2.27 (2.10–2.46)
Rosner et al,^40^ 2019		5.9 (2.1–16.0)	0.39 (0.18–0.82)	2.52 (1.81–3.50)
Sevene et al,^41^ 2020		0.9 (0.3–2.6)	1.01 (0.90–1.13)	0.94 (0.37–2.42)
Sutton et al,^43^ 2020		2.9 (2.6–3.2)	0.67 (0.62–0.70)	2.11 (1.98–2.24)

Diagnostic threshold: sBP 130 mmHg or dBP 80 mmHg				
Pooled	<130/80 mmHg (normal BP + elevated BP)	3.6 (2.4–5.6); *I*^2^=99% (1.4–9.9)	0.66 (0.51–0.78)	2.93 (2.32–3.67)
Bellad et al,^29^ 2020		0.8 (0.6–0.9)	0.95 (0.94–0.97)	2.64 (2.00–3.48)
Bello et al,^30^ 2021		8.4 (7.9–8.9)	0.44 (0.42–0.46)	4.25 (4.14–4.37)
Darwin et al,^8^ 2021		4.7 (3.5–6.3)	0.56 (0.47–0.66)	2.98 (2.53–3.50)
Duffy et al,^31^ 2021		2.1 (2.0–2.2)	0.79 (0.77–0.80)	1.73 (1.69–1.77)
Greenberg et al,^9^ 2021		4.3 (3.9–4.8)	0.67 (0.64–0.70)	3.37 (3.12–3.64)
Huai et al,^35^ 2021		3.1 (2.0–5.0)	0.36 (0.23–0.55)	1.35 (1.24–1.46)
McLaren et al,^37^ 2021		2.6 (1.7–4.1)	0.46 (0.32–0.68)	1.79 (1.43–2.25)
Qureshi et al,^39^ 2020		6.0 (4.7–7.5)	0.68 (0.62–0.75)	4.71 (3.96–5.61)
Reddy et al,^13^ 2020		3.6 (3.0–4.3)	0.73 (0.69–0.79)	2.79 (2.46–3.16)
Sabol et al,^14^ 2021		4.5 (3.6–5.5)	0.55 (0.49–0.63)	3.15 (2.76–3.60)
Sevene et al,^41^ 2020		0.5 (0.1–3.3)	1.03 (0.98–1.10)	0.46 (0.07–3.25)
Sutton et al,^43^ 2020		4.2 (3.8–4.7)	0.73 (0.70–0.76)	3.67 (3.35–4.02)

Diagnostic threshold: sBP 140 mmHg or dBP 90 mmHg				
Pooled	<140/90 mmHg (normal BP + elevated BP + stage 1 HTN)	5.2 (4.1–6.5); I^2^=94% (1.7–15.10)	0.80 (0.69, 0.89)	5.95 (4.17, 8.12)
Bellad et al,^29^ 2020		4.8 (3.3–6.9)	0.98 (0.97–0.99)	6.38 (3.77–10.79)
Bello et al,^30^ 2021		8.3 (7.8–8.8)	0.73 (0.71–0.74)	7.66 (7.27–8.07)
Darwin et al,^8^ 2021		4.3 (3.2–5.8)	0.71 (0.63–0.80)	3.53 (2.80–4.46)
Greenberg et al,^9^ 2021		5.6 (5.0–6.3)	0.75 (0.72–0.78)	5.49 (4.93–6.12)
Huai et al,^35^ 2021		2.9 (2.0–4.1)	0.45 (0.34–0.60)	1.61 (1.43–1.81)
McLaren et al,^37^ 2021		2.0 (1.4–2.9)	0.84 (0.73–0.96)	2.61 (1.41–4.83)
Qureshi et al,^39^ 2020		12.7 (9.6–16.8)	0.88 (0.84–0.92)	18.82 (12.28–28.83)
Reddy et al,^13^ 2020		6.5 (4.8–8.7)	0.93 (0.90–0.95)	7.22 (5.17–10.07)
Rosner et al,^40^ 2019		4.8 (1.7–13.4)	0.82 (0.64–1.05)	4.69 (1.76–12.52)
Sabol et al,^14^ 2021		5.3 (4.3–6.5)	0.67 (0.61–0.73)	5.24 (4.31–6.36)
Sevene et al,^41^ 2020		1.9 (0.3–13.0)	0.99 (0.93–1.04)	1.97 (0.26–14.70)
Sutton et al,^43^ 2020		5.5 (4.9–6.1)	0.79 (0.77–0.82)	5.84 (5.17–6.60)

*ACC-AHA*, American College of Cardiology-American Heart Association; *BP*, blood pressure; *CI*, confidence interval; −*LR*, negative likelihood ratio; *+LR*, positive likelihood ratio; *PI*, prediction interval; *RR*, relative risk.

*Slade. The diagnostic accuracy of American College of Cardiology-American Heart Association blood pressure categories for identification of women and babies at risk. Am J Obstet Gynecol 2023*.

Table 3 shows the associations and diagnostic test properties for BP cutoffs and other main and secondary outcomes; for details of individual studies, see Tables S4 to 15. There was a general pattern of significant associations between higher BP values measured before <20 weeks gestation and adverse pregnancy outcomes, usually with a dose-response relationship evident, and all adverse outcomes evaluated were associated with stage 2 hypertension. However, as for the outcome of preeclampsia (Table 2), the BP cutoffs for elevated BP or stage 1 hypertension was not useful in ruling out or ruling in adverse pregnancy outcomes. Although a BP <140/90 mmHg could not rule out adverse outcomes, a diagnosis of stage 2 hypertension was useful in identifying women at particular risk for eclampsia, stroke, and maternal ICU admission; these outcomes were informed primarily by trials from less developed countries with community-ascertained outcomes.^29^^,^^39^^,^^41^

**Table 3:** Pooled relative risk and mean likelihood ratios for other main and secondary outcomes according to BP cutoffs at <20 weeks gestation based on ACC-AHA BP categories

Outcome	Ref	Number of studies	Number of women	RR (95% CI); *I*^2^ (95% PI)	−LR (95% CI)	+LR (95% CI)
Diagnostic threshold: sBP 120 mmHg (with dBP <80 mmHg)						
Serious maternal complications		6^a^	51,095	1.2 (0.9–1.8); *I*^2^=50%, (0.4–3.7)	0.97 (0.89–1.04)	1.19 (0.86–1.62)
Preterm birth		8	100,663	1.2 (1.0–1.5); I^2^=94%, (0.6–2.5)	0.94 (0.89–1.00)	1.21 (1.01–1.43)
Perinatal death		5	51,699	1.4 (1.2–1.6); *I*^2^=11%, (1.1–1.7)	0.94 (0.87–0.97)	1.34 (1.17–1.54)
Stillbirth		6	51,095	1.2 (0.9–1.8); *I*^2^=51%, (0.4–3.7)	0.97 (0.89–1.04)	1.18 (0.86–1.62)
Newborn death		5	50,410	1.4 (1.2–1.7); *I*^2^=0%, (1.0–1.9)	0.94 (0.88–0.98)	1.35 (1.14–1.59)
SGA		5	68,908	1.0 (0.9–1.1); *I*^2^=79%, (0.7–1.5)	1.00 (0.96–1.04)	1.00 (0.91–1.09)
NICU admission		3	34,401	1.5 (1.2–1.8); *I*^2^=45%, (0.2–10.15)	0.87 (0.82–0.92)	1.36 (1.21–1.52)
Eclampsia		4	38,586	2.2 (0.8–6.5); *I*^2^=40%, (UTD)	0.89 (0.62–1.00)	1.72 (0.97–2.60)
Stroke		3	19,387	2.1 (0.7–6.2); *I*^2^=63%, (UTD)	0.88 (0.65–1.03)	2.08 (0.74–4.39)
Maternal ICU admission		4	38,187	1.5 (1.1–2.0); *I*^2^=0%, (0.8–2.9)	0.86 (0.79–0.92)	2.02 (1.31–3.06)
Placental abruption		4	37,379	1.1 (0.9–1.3); *I*^2^=0%, (0.7–1.6)	0.98 (0.93–1.03)	1.05 (0.90–1.22)
Cesarean		4	19,752	1.3 (1.2–1.4); *I*^2^=0%, (1.0–1.6),	0.96 (0.93–0.98)	1.27 (1.13–1.45)
**Diagnostic threshold: sBP 130 mmHg or dBP 80 mmHg**						
Serious mat complications		8	357,659	1.6 (1.3–1.9); *I*^2^=29%, (1.1–2.2)	0.93 (0.89–0.96)	1.47 (1.18–1.69)
Preterm birth		13	514,632	1.5 (1.3–1.8); *I*^2^=98%, (0.7–3.3)	0.92 (0.84–0.96)	1.49 (1.24–1.77
Perinatal death		5	51,699	1.6 (1.4–1.9); *I*^2^=0%, (1.2–2.1)	0.95 (0.92–0.97)	1.59 (1.29–1.94)
Stillbirth		8	357,659	1.6 (1.3–1.9); *I*^2^=29%, (1.1–2.2)	0.93 (0.89–0.96)	1.47 (1.28–1.69)
Newborn death		5	50,410	1.6 (1.3–2.1); *I*^2^=14%, (1.0–2.7)	0.96 (0.93–0.98)	1.57 (1.19–2.05)
SGA		10	497,236	1.1 (1.0–1.2); *I*^2^=78%, (0.8–1.4)	0.99 (0.96–1.00)	1.06 (0.99–1.14)
NICU admission		6	462,080	1.4 (1.2–1.7); *I*^2^=96%, (1.1–1.8)	0.92 (0.86–0.97)	1.39 (1.11–1.72)
Eclampsia		5	342,275	2.5 (1.1–5.4); *I*^2^=66%, (0.1–48.6)	0.85 (0.67–0.96)	2.47 (1.39–4.32)
Stroke		4	323,076	2.1 (0.8–5.6); *I*^2^=76%, (0.0–105.9)	0.86 (0.84–0.91)	2.80 (1.40–5.20)
Maternal ICU admission		4	38,187	2.3 (1.7–3.1); *I*^2^=0%, (1.1–4.6)	0.93 (0.84–0.99)	1.78 (1.28–2.35)
Placental abruption		8	344,717	1.1 (1.0–1.2); *I*^2^=0%, (1.0–1.3)	0.97 (0.94–0.99)	1.10 (1.04–1.17)
Cesarean delivery		7	447,431	1.3 (1.1–1.6); *I*^2^=99%, (1.0–1.9)	0.95 (0.90–0.97)	1.45 (1.26–1.69)

Diagnostic threshold: sBP 140 mmHg or dBP 90 mmHg						
Serious maternal complications		8	54,347	3.5 (2.5–4.9); *I*^2^=0%, (2.3–5.3)	0.93 (0.86–0.97)	3.33 (2.23–4.80)
Preterm birth		12	210,923	2.3 (2.0–2.6); *I*^2^=82%, (1.1–4.6)	0.95 (0.89–0.98)	2.51 (2.04–3.06)
Perinatal death		5	51,699	2.4 (1.8–3.1); *I*^2^=0%, (1.5–3.8)	0.98 (0.95–0.99)	2.54 (1.76–3.56)
Stillbirth		8	54,347	3.5 (2.5–4.0); *I*^2^=0%, (2.3–5.3)	0.93 (0.86–0.97)	3.26 (2.23–4.80)
Newborn death		5	50,410	1.9 (1.1–3.4); *I*^2^=0%, (0.4–9.4)	0.99 (0.97–1.00)	2.04 (1.00–3.73)
SGA		9	193,527	1.3 (1.2–1.4); *I*^2^=47%, (1.0–1.6)	0.98 (0.96–0.99)	1.32 (1.17–1.48)
NICU admission		6	158,768	1.8 (1.3–2.4); *I*^2^=93%, (0.4–9.0)	0.95 (0.91–0.99)	1.91 (1.18–3.15)
Eclampsia		4	38,586	7.3 (3.8–14.3); *I*^2^=0%, (0.1–560.9)	0.89 (0.69–0.96)	7.46 (4.16–13.3)
Stroke		3	19,387	8.6 (1.5–50.2); *I*^2^=0%, (UTD)	0.90 (0.77–0.98)	12.7 (2.11–41.10)
Maternal ICU admission		4	38,187	4.0 (2.8–5.6); *I*^2^=0%, (1.9–8.4)	0.91 (0.84–0.96)	6.34 (3.55–10.50)
Placental abruption		6	40,631	1.4 (1.0–1.9); *I*^2^=0%, (0.8–2.3)	0.97 (0.91–1.00)	1.40 (1.04–1.85)
Cesarean delivery		7	144,119	1.7 (1.6–1.8); *I*^2^=27%, (1.5–1.9)	0.97 (0.93–0.98)	2.30 (2.06–2.55)

*ACC-AHA*, American College of Cardiology-American Heart Association; *BP*, blood pressure; *CI*, confidence interval; *dBP*, diastolic blood pressure; *ICU*, intensive care unit; *NICU*, neonatal intensive care unit; *PI*, prediction interval; *PPH*, postpartum hemorrhage; *sBP*, systolic blood pressure; *SGA*, small for gestational age; *UTD*, unable to determine.

*Slade. The diagnostic accuracy of American College of Cardiology-American Heart Association blood pressure categories for identification of women and babies at risk. Am J Obstet Gynecol 2023*.

a One additional study was not included, because complications such as gestational diabetes mellitus, cesarean delivery, and all forms of pregnancy hypertension were part of the composite.^39^

Other secondary maternal or neonatal outcomes that were reported on by the included studies, but at event rates too low for meaningful comparisons, were maternal postpartum hemorrhage (n=2/23), maternal death (n=3/23), HELLP syndrome (n=3/23), pulmonary edema (n=1/23 studies), acute kidney injury (n=1/23 studies), miscarriage (n=2/23 studies), newborn respiratory support (n=1/23), and neonatal seizures (n=2/23). The following maternal morbidities were not reported at all: intubation or ventilation, cortical blindness, retinal detachment, or liver capsule hematoma.

## Discussion

### Main findings

This systematic review demonstrated that higher BPs before 20 weeks gestation is associated with a greater risk for subsequent preeclampsia and most adverse pregnancy outcomes. In general, and in comparison with normal BP according to the ACC/AHA criteria, the risk for preeclampsia (and some other adverse outcomes) was greatest among participants with stage 2 hypertension, followed by those with stage 1 hypertension, and then by those with elevated BP. However, when the relevant cutoffs were examined, only the 140/90 mmHg cutoff for stage 2 hypertension when measured before 20 weeks gestation could accurately identify women at increased risk; our findings were similar for other adverse pregnancy outcomes. Neither the lower ACC/AHA BP cutoffs of 120/80 mmHg and 130/80 mmHg nor the currently used 140/90 mmHg cutoff demonstrated any ability to rule out preeclampsia or other adverse pregnancy outcomes. Taken together, these findings suggest that the definition of chronic hypertension in pregnancy, consistent with stage 2 hypertension, should not be revised.

### Comparison with the literature

Our findings are consistent with literature reporting that chronic hypertension (ie, stage 2 hypertension) is a major risk factor for superimposed preeclampsia and other adverse pregnancy outcomes.^48^ By systematic review of 81 observational studies, chronic hypertension was associated with higher odds of developing preeclampsia (∼5-fold), maternal mortality (∼5-fold), cesarean delivery (∼2-fold), stillbirth (∼2-fold), preterm birth (∼2-fold), and SGA infants (∼2-fold).^49^

Our findings are consistent with the published association between higher BP levels (below 140/90 mmHg) and subsequent preeclampsia, a BP ≥140/90 mmHg being 1 of the criteria along with proteinuria or another manifestations of maternal end-organ dysfunction according to ACOG.^50^ A recent systematic review of prediction models for gestational hypertension and preeclampsia included 40 different models with varying ability to predict preeclampsia. Of 31 predictors, 17 were related to BP as a continuous measure in 12 studies, as the mean arterial pressure, as diastolic BP in 3, and as systolic BP in 2^51^; only BMI was included more frequently in 19 of 40 models.

### Strengths and limitations

Strengths of our analysis include inclusion of more than 700,000 women in diverse locations, from countries in Australasia, South America, South Asia, Africa, and East Asia, although most publications reported on women in America. The included studies reported on multiple populations at different risks for preeclampsia–1 at high risk, 3 using only nulliparous participants, and 7 using other low-risk women.

Although multiple pregnancy was not specifically addressed in this review, only 4 studies included women with multifetal gestations,^29^^,^^33^^,^^39^^,^^41^ and multiple pregnancy represented only a very small proportion of women.

Limitations included variable definitions of preeclampsia and variable preeclampsia incidence. We had different subsets of studies for evaluation of each ACC-AHA BP level, because only 7 studies reported on all 4 BP categories.^9^^,^^11^^,^^13^^,^^29^^,^^39^^,^^41^^,^^43^ Many studies combined categories to investigate the association between different BP thresholds instead of investigating the categories individually, which limited our analyses.^10^^,^^14^^,^^31^^,^^32^^,^^35^, ^36^, ^37^, ^38^^,^^40^^,^^42^^,^^44^^,^^45^ Although severe preeclampsia was reported in 13 of 23 studies, there was considerable heterogeneity between definitions, often specific to particular study authors; this prohibited use of severe preeclampsia as an outcome for meta-analysis.

We analyzed aggregate data sets, which precluded full exploration of interactions between BP level and other maternal or pregnancy characteristics, as well as use of BP as a continuous variable. Although BP is not used this way in clinical practice, multivariable models suggest that it has independent predictive power for preeclampsia when used in this fashion.^52^ There were limited possible comparisons for stage 2 hypertension with lower BP categories, and only 1 study reported stage 2 hypertension by severity, making impossible an analysis by severity of existing chronic hypertension.

The influence of demographic factors, particularly overweight and obesity, that compound the risk for adverse pregnancy outcomes is unclear from this analysis. Because this was not an individual participant data meta-analysis, we could not adjust for baseline risk factors; however, given that adjustment factors (eg, obesity) are confounders in the relationship between BP and adverse outcome, the unadjusted analyses would likely have overestimated the relationship between BP and adverse outcome.

Previous studies have not yet addressed whether treatment of hypertension at thresholds <140/90 mmHg in pregnancy is of benefit. However, the Control of Hypertension In Pregnancy Study (CHIPS) trial showed that control of BP (to a diastolic of 85 mmHg) reduced the incidence of severe maternal hypertension and severe features of preeclampsia (ie, platelet count <100×10^9^/L and elevated liver enzymes with symptoms).^53^ In addition, the recent Control of Hypertension And Pregnancy (CHAP) trial showed a reduction in preeclampsia with severe features and medically indicated delivery at <35+0 weeks.^54^ In neither CHIPS nor CHAP was BP control associated with an increase in SGA neonates. Treatment for BPs below the current threshold for hypertension is not suggested by any major international guideline.^55^, ^56^, ^57^ Many studies in this review excluded women on antihypertensives for any other indication throughout their pregnancy.

### Perspectives

BP measurements taken early in pregnancy contributes to delineating the most appropriate model of surveillance and care throughout pregnancy. Compared with women considered to have normal BP, women with chronic hypertension are seen more frequently and have ultrasound surveillance for fetal growth.^56^ Adjusting the threshold of chronic hypertension in pregnancy (to a level below 140/90 mmHg) based only on the association with poor outcomes and poor diagnostic test properties for their identification would significantly increase the workload associated with surveillance of these women without tangible benefits. Although also anticipated to increase costs, an economic analysis of such reclassifications still has to be undertaken.^58^

These data highlight the importance of ongoing monitoring of BP throughout pregnancy, because even normal BP (<120/80 mmHg) before 20 weeks gestation is not useful in ruling out the development of preeclampsia or related adverse pregnancy outcomes after 20 weeks.

Progress in the prediction of preeclampsia or other adverse outcomes from BP measurements is most likely to come from efforts focused on BP levels as a continuous variable or on other BP features (such as variability), specifically as part of multivariable models that include other aspects of maternal history, ultrasonographic assessment, and angiogenic markers.^59^

### Conclusion and implications

This systematic review of publications examining the relationship between the 2017 ACC-AHA BP criteria and pregnancy outcomes has found no evidence that lowering the diagnostic threshold for chronic hypertension in pregnancy to below 140/90 mmHg would assist clinicians in identifying women more likely to develop preeclampsia or other adverse pregnancy outcomes.

## Figures and Tables

**Table:** 

	Title	Page number
**Figures**		
Figure S1	PRISMA flow diagram for study inclusion and exclusion	See separate
**Tables**		
Table S1	Details of QUADAS-2 assessment for included studies	3-6
Table S2	Study characteristics arranged by strength of association with preeclampsia by highest BP category reported	7-8
Table S3	ACC/AHA blood pressure categories reported by each study for measurements taken <20 weeks	9
Table S4	Sensitivity and specificity of ACC/AHA BP categories for preeclampsia	10
	***Other main outcomes***	
Table S5	Association of ACC/AHA BP levels at <20 weeks and serious maternal complications	11
Table S6	Sensitivity and specificity of ACC/AHA BP categories for serious maternal outcomes	12
Table S7	Association of ACC/AHA BP levels at <20 weeks and preterm birth	13
Table S8	Sensitivity and specificity of ACC/AHA BP categories for preterm birth	14
Table S9	Association of ACC/AHA BP levels at <20 weeks and perinatal death	15
Table S10	Sensitivity and specificity of ACC/AHA BP categories for preterm birth	16
Table S11	Association of ACC/AHA BP levels at <20 weeks and stillbirth	17
Table S12	Sensitivity and specificity of ACC/AHA BP categories for stillbirth	18
Table S13	Association of ACC/AHA BP levels at <20 weeks and newborn death	19
Table S14	Sensitivity and specificity of ACC/AHA BP categories for newborn death	20
Table S15	Association of ACC/AHA BP levels at <20 weeks and SGA infants	21
Table S16	Sensitivity and specificity of ACC/AHA BP categories for SGA infants	22
Table S17	Association of ACC/AHA BP levels at <20 weeks and NICU admission	23
Table S18	Sensitivity and specificity of ACC/AHA BP categories for NICU admission	24
	***Other secondary outcomes***	
Table S19	Association of ACC/AHA BP levels at <20 weeks and eclampsia	25
Table S20	Sensitivity and specificity of ACC/AHA BP categories for eclampsia	26
Table S21	Association of ACC/AHA BP levels at <20 weeks and stroke	27
Table S22	Sensitivity and specificity of ACC/AHA BP categories for stroke	28
Table S23	Association of ACC/AHA BP levels at <20 weeks and maternal admission to intensive care	29
Table S24	Sensitivity and specificity of ACC/AHA BP categories for maternal admission to intensive care	30
Table S25	Association of ACC/AHA BP levels at <20 weeks and placental abruption	31
Table S26	Sensitivity and specificity of ACC/AHA BP categories for placental abruption	32
Table S27	Association of ACC/AHA BP levels at <20 weeks and Cesarean	33
Table S28	Sensitivity and specificity of ACC/AHA BP categories for Cesarean	34

*Slade. The diagnostic accuracy of American College of Cardiology-American Heart Association blood pressure categories for identification of women and babies at risk. Am J Obstet Gynecol 2023*.

**Table S1:** Details of QUADAS-2 tool for risk-of-bias assessment of for included studies

Paper	QUADAS domain					Overall
	1A	1B	2	3	4	
^29^	Low	Low	Low - thorough description, trained staff, semi-automated machine	Low	Low	Low
^30^	Low – singleton only, excluded only if no BP <20 weeks or medical comorbidity requiring antihypertensive use for indication other than lowering BP	Low	Unclear – no description of measurement	Unclear	Low	Unclear
^8^	Low – excluded lost to follow up and women given aspirin	Low	Low – single measurement, 5 mins rest, trained staff, automated sphygmomanometer, appropriately sized cuff	Low	Low	Low
^31^	Low - excluded <18 years old or fewer than 2 antenatal visits before 20 weeks of gestation	Low	Unclear – no description of measurement	Unclear	High	High
^32^	High - excluded chronic diseases including thrombophilia, heart disease, neuropathy, neurodegenerative disease, cancer or autoimmune disease, ART, fetal anomaly or aneuploidy, or delivery at different institution	Low	Unclear –aneroid machine but no other description of measurements	Unclear	Low	High
^9^	High – excluded maternal comorbidities – SLE, pre-existing diabetes, lack of maternal weight recorded or gestational weight gain, initiation of antihypertensive medication < 20 weeks without diagnosis of HDP	Low	Unclear – no description of measurement	Unclear	Low	High
^33^	High – nulliparous only. Excluded history of 3 or more spontaneous abortions, current pregnancy complicated by aneuploidy or fetal malformation, assisted reproduction with a donor oocyte, multifetal reduction or plans to terminate the pregnancy	Low	Low – aneroid sphygmomanometer, trained staff, sized cuff, rested and seated, repeated x 3	Low	Low	High
^10^	High – chronic hypertension, multiple pregnancy Included only those with pre-existing diabetes or history of previous PET	Low	Low – mercury sphygmomanometer, seated 10 mins, trained study staff, cuff to size	Low	Low	High
^52^	High – excluded multiple pregnancy, multiparous women, chronic hypertension, missing variables – large proportion excluded	Low	Unclear – booking visit, right arm but no other specifications	Unclear	Low	High
^11^	High – excluded PET late pregnancy, birth defects, tobacco and alcohol use, chronic hypertension based on criteria <20 weeks, missing covariates and those who delivered more than once in the study period	Low	Low – seated, automated sphygmomanometer, repeated and averaged	Low	High	High
^35^	High – excluded those with missing BP data or chronic hypertension	Low	Low – mercury sphygmomanometer, trained staff, seated, resting, multiple measurements averaged	Low	Low	High
^36^	High – excluded multiple pregnancy, stillbirth, diabetes mellitus, thyroid disease, renal disease, chronic hypertension, PCOS, incomplete records	Low	Low – trained staff, mercury sphygmomanometer, upright, right arm, repeated and averaged	Low	Low	High
^37^	Low – excluded multiples, anomalies and lost to follow up <20 weeks	Low	Low – manual device, described technique 15 mins seated rest	Low	High – 1933 women met inclusion criteria but only 252 included overall∗	High
^38^	High – excluded multiple pregnancy, miscarriage, stillbirth, chronic hypertension, missing data	Low	Low – automated device, described measurement technique arm at heart level	Low	Low	High
^39^	Low	Low	Low - thorough description, trained staff, semi-automated machine	Low	Low	Low
^13^	Low – multiple pregnancy, fetal anomalies and no BP measurements in specified gestations	Low	Unclear – trained staff, doesn’t specify which type of sphygmomanometer	Unclear	Low	Unclear
^3^	Low – excluded only multiple pregnancy and those entering care >12 weeks	Low	Unclear – BP left arm seated position but no other comments	Unclear	Low	Unclear
^14^	Low - excluded multiples, delivery <20 weeks, missing prenatal records or BP data	Low	Unclear – not described	Unclear	Low	Unclear
^41^	Low	Low	Low - thorough description, trained staff, semi-automated machine	Low	Low	Low
^43^	Low – excluded multiple pregnancy, incomplete records, second delivery in study period or miscarriage	Low	High – records from multiple practices, not regulated by research protocol	High	Low	High
^42^	High – cut off was 135/85, excluded chronic hypertension above that, renal disease, diabetes or anyone with proteinuria on dipstick	Low	Low – mercury sphygmomanometer, study staff, seated and rested	Low	Low	High
^44^	High – excluded multiples, renal disease, stillbirth, hypertension, diabetes, thyroid dysfunction in early pregnancy	Low	Low – trained nurses, mercury sphygmomanometer, comfortable seat, 2 measurements	Low	Low	High
^45^	High – excluded <2 BP measurements <20 weeks, chronic hypertension, cardiovascular or hepatic disease, diabetes, autoimmune disease, multiple gestation or fetal anomaly	Low	Low – automatic sphygmomanometer, at least 2 measurements, seated position, left arm, resting	Low	Low	High

ACC (American College of Cardiology), AHA (American Heart Association), ART (assisted reproductive technologies), BP (blood pressure), HDP – hypertensive disorder of pregnancy, PCOS (polycystic ovarian syndrome), PET (preeclampsia), SLE (systemic lupus erythematosus)

*Slade. The diagnostic accuracy of American College of Cardiology-American Heart Association blood pressure categories for identification of women and babies at risk. Am J Obstet Gynecol 2023*.

∗ For every patient placed into either the ACC-AHA group or the ACOG group, the next patient with normal blood pressures was placed in the control group.

**Table S2:** Study characteristics arranged by strength of association with preeclampsia by highest BP category reported

	QUADAS2 summary	Av age	Av BMI	Ethnicity	MMR of country (per 100 000 live births)	PET definition	PET prevalence	BP device	BP gestation (weeks)	Proportion nulliparous	BP type	BP type – routine or study	Cohort	Total number participants
^39^	Low	28	NS	S Asian	289	ISSHP 2018	2.5%	Automated	<20	23%	Av of 2 or 3 in 1 visit	Study∗	Unselected	10839
^30^	Low	30	27	52% H	18	ICD codes	3.5%	NS	<20	41%	=/>2 visits in 30 days	Routine	Unselected	132032
^13^	Low	31	30	Mixed	6	ACOG 2019	2.9%	Mercury	Multiple	40%	Single	Routine	Unselected	18243
^9^	High	25% >35	25% >30	56% NHW	18	ACOG 2013	6.3%	NS	<20	43%	=/> 2 visits	Routine	Low risk	18801
^14^	Low	28	36% >30	Mixed	18	ACOG 2019	3.1%	NS	<20	41%	Single	Routine	Unselected	30000
^40^	Low	34	23	NR	18	ND	3.4%	NS	<20	54%	Single	Routine	Unselected	377
^43^	High	30	27	70% NHW	19	ICD codes	6.9%	NS	<20	44%	=/> 2 visits	Routine	Unselected	18162
^29^	Low	23	NS	S Asian	145	ISSHP 2018	13.0%	Automated	<20	36%	Av 2 or 3 in 1 visit	Study∗	Unselected	6067
^52^	High	6% >35	9% >30	Asian	36	ACOG 2013	2.1%	Mercury	12	100%	Single ##	Routine	Nulliparous only	58054
^8^	Low	30	26	51% NHB	15	ACOG 2019	5.4%	Automated	11-14	74%	Single	Study	Unselected	2976
^32^	High	24.2	24	NR	83		7.8%	Anaeroid	<20	42%	=/> 2, at least 4 hours apart	Routine	Low risk	293
^36^^ˆ^	High	39	22	Asian	36	ACOG 2019	1.7%	Mercury	<20		Av of 3 in 1 visit	Study	Low risk	6284
^37^	High	30	26	Mixed	18	ACOG 2013	30%	NS	<20	35%	Single	Routine	Unselected	252
^35^Δδφ	High	32	25	Asian	29	ACOG 2013	18.1%	Mercury	<20	36%	Av of 2 in 1 visit	Study	Low risk	838
^44^	High	NS	10% >30	Asian	29	ISSHP 2018	8.2%	Mercury	<20	78%	Av of 2 in 1 visit	Routine	Low risk	47874
^31^	High	30	26	52% H	19	ICD codes	3.7%	NS	<20	29%	Single	Routine	Unselected	303689
^42^δφ	Highδ	21	23	50% NHB	12	ACOG 2003	7.8%	Mercury	(+ <20)	100%	Single	Study	LRN only	2947
^45^	High	32	21	Asian	97	ACOG 2013	3.0%	Automated	<12	50%	=/> 2 visits	Routine	Low risk	2039
^10^δφ	High	25	28	58% NHB	12	ACOG 2003	17.9%	Mercury	(+ <20)		Single	Study	High risk	1020
^33^	High	27	26	61% NHW	14	ACOG 2013	8.3%	Anaeroid	11-12	100%	Single	Study	Nulliparous only	8899
^41^^ˆ^	Low	23	NS	African	140	ISSHP 2018	0.9%	Automated	<20	31%	Av of 2 or 3 in 1 visit	Study∗	Unselected	4163

ACOG (American College of Obstetricians and Gynaecologists), Av (average), BP (blood pressure), H (Hispanic), ICD (International Classification of Disease), ISSHP (International Society for Hypertension in Pregnancy), LRN (low risk nulliparous) ND (not defined), NHB (non Hispanic Black), NS (not stated), OPC (observational prospective controlled), ORC (observational retrospective controlled), PET (preeclampsia), S Asian (South Asian), SA (secondary analysis)ˆ includes unpublished data.

*Slade. The diagnostic accuracy of American College of Cardiology-American Heart Association blood pressure categories for identification of women and babies at risk. Am J Obstet Gynecol 2023*.

## But used lower if multiple measurements done

δ Randomised trial

ϕ Aspirin trial

∗ Study BP values for CLIP trials.

**Table S3:** ACC/AHA blood pressure categories reported by each study

Paper	Normal	Elevated	Stage 1 hypertension	Stage 2 hypertension
Bellad				X
Bello				X
Darwin				X
Duffy				X
Gonzalez-Valencia				
Hauspurg 19				
Hauspurg 18				
He				
Hu∗				X
Huai				
Li				
McLaren				X
Nagao∗				
Qureshi				X
Reddy				X
Rosner				X
Sabol				X
Sevene				X
Suttton 2020				X
Sutton 2018				
Wu				
Yang				

*Slade. The diagnostic accuracy of American College of Cardiology-American Heart Association blood pressure categories for identification of women and babies at risk. Am J Obstet Gynecol 2023*.

**Table S4:** Sensitivity and Specificity of ACC/AHA BP categories for Preeclampsia

		COMPARATOR	
BP measured at <20 weeks	Ref	Sensitivity	Specificity
**‘ELEVATED BP’ (as a new cut-off)**			
**Pooled**	**Normal (<120/80mmHg)**	**0.42 [0.25, 0.60]**	**0.83 [0.74, 0.89]**
Bellad		0.11 [0.09, 0.13]	0.95 [0.95, 0.95]
Greenberg		0.56 [0.53, 0.58]	0.74 [0.73, 0.74]
Qureshi		0.47 [0.42, 0.53]	0.86 [0.86, 0.87]
Reddy		0.57 [0.53, 0.62]	0.75 [0.74, 0.75]
Rosner		0.72 [0.49, 0.88]	0.71 [0.66, 0.76]
Sevene		0.11 [0.04, 0.25]	0.88 [0.86, 0.90]
Sutton 2020		0.49 [0.46, 0.52]	0.77 [0.76, 0.77]
**‘STAGE 1 HYPERTENSION’ (as a new cut-off)**			
**Pooled**	**Normal + Elevated BP (<130/80mmHg)**	**0.44 [0.29, 0.61]**	**0.85 [0.74, 0.92]**
Bellad		0.07 [0.06, 0.09]	0.97 [0.97, 0.98]
Bello		0.63 [0.61, 0.64]	0.85 [0.85, 0.85]
Darwin		0.54 [0.47, 0.62]	0.82 [0.80, 0.83]
Duffy		0.39 [0.38, 0.40]	0.77 [0.77, 0.77]
Huai		0.88 [0.81, 0.92]	0.35 [0.32, 0.39]
Greenberg		0.42 [0.39, 0.44]	0.88 [0.87, 0.88]
McLaren		0.72 [0.61, 0.81]	0.60 [0.52, 0.67]
Qureshi		0.37 [0.31, 0.43]	0.92 [0.92, 0.93]
Reddy		0.36 [0.31, 0.43]	0.87 [0.87, 0.88]
Sabol		0.54 [0.49, 0.60]	0.83 [0.81, 0.84]
Sevene		0.03 [0.00, 0.14]	0.94 [0.92, 0.95]
Sutton 2020		0.34 [0.31, 0.37]	0.91 [0.90, 0.91]
**‘STAGE 2 HYPERTENSION’ (existing cut-off)**			
**Pooled**	**Normal + Elevated BP + Stage 1 HTN (<140/90mmHg)**	**0.22 [0.13, 0.36]**	**0.96 [0.92, 0.98]**
Bellad		0.03 [0.02, 0.04]	1.00 [0.99, 1.00]
Bello		0.30 [0.29, 0.31]	0.96 [0.96, 0.96]
Darwin		0.36 [0.29, 0.44]	0.90 [0.89, 0.91]
Greenberg		0.29 [0.27, 0.32]	0.95 [0.94, 0.95]
Huai		0.76 [0.69, 0.82]	0.53 [0.49, 0.56]
McLaren		0.24 [0.16, 0.34]	0.91 [0.86, 0.94]
Qureshi		0.12 [0.09, 0.17]	0.99 [0.99, 1.00]
Reddy		0.08 [0.06, 0.11]	0.99 [0.99, 0.99]
Sabol		0.38 [0.33, 0.44]	0.93 [0.92, 0.94]
Rosner		0.22 [0.09, 0.45]	0.95 [0.93, 0.97]
Sevene		0.03 [0.00, 0.14]	0.99 [0.98, 0.99]
Sutton 2020		0.24 [0.22, 0.27]	0.96 [0.96, 0.96]

*Slade. The diagnostic accuracy of American College of Cardiology-American Heart Association blood pressure categories for identification of women and babies at risk. Am J Obstet Gynecol 2023*.

**Table S5:** Pooled relative risk and mean likelihood ratios for serious maternal complications, according to BP cut-offs at <20 weeks, determined by ACC/AHA BP categories

		COMPARATOR		
BP measured at <20 weeks	Ref	RR [95% CI] I^2^, (95% PI)	-LR	+LR
**Diagnostic threshold: sBP 120mmHg (with dBP <80mmHg)**				
**Pooled**	**Normal (<120/80mmHg)**	**1.2 [0.9, 1.8] I^2^=51%, (0.4, 3.7)**	**0.97 [0.89, 1.04]**	**1.19 [0.86, 1.62]**
Bellad		1.5 [1.0, 2.2]	0.97 [0.95, 1.00]	1.49 [1.02, 2.17]
Qureshi		1.6 [1.3, 2.1]	0.91 [0.86, 0.96]	1.55 [1.27, 1.89]
Reddy		0.7 [0.4, 1.3]	1.07 [0.96, 1.20]	0.79 [0.52, 1.22]
Rosner		0.4 [0.0, 9.3]	1.21 [0.72, 2.01]	0.54 [0.04, 6.79]
Sevene		0.4 [0.1, 2.8]	1.05 [0.94, 1.18]	0.57 [0.12, 2.73]
Sutton 2020		2.3 [0.5, 10.1]	0.75 [0.41, 1.38]	1.76 [0.80, 3.86]
**Diagnostic threshold: sBP 130mmHg or dBP 80mmHg**				

**Pooled**	**Normal + Elevated BP (<130/80mmHg)**	**1.6 [1.3, 1.9] I^2^=29%,(1.1, 2.2)**	**0.93 [0.89, 0.96]**	**1.47 [1.28, 1.69]**

Bellad		1.8 [1.1, 2.9]	0.98 [0.95, 1.00]	1.81 [1.12, 2.92]
Duffy		1.5 [1.3, 1.6]	0.90 [0.87, 0.94]	1.31 [1.21, 1.43]
McLaren		5.0 [0.6, 42.2]	0.33 [0.05, 1.97]	1.69 [1.16, 2.48]
Qureshi		1.8 [1.4, 2.4]	0.93 [0.89, 0.97]	1.75 [1.36, 2.27]
Reddy		0.7 [0.3, 1.5]	1.04 [0.96, 1.12]	0.75 [0.39, 1.45]
Sabol		2.9 [1.1, 7.8]	0.71 [0.46, 1.10]	2.10 [1.20, 3.67]
Sevene		0.8 [0.1, 5.7]	1.01 [0.92, 1.11]	0.79 [0.11, 5.45]
Sutton 2020		1.4 [0.2, 11.2]	0.96 [0.71, 1.30]	1.30 [0.21, 8.00]
**Diagnostic threshold: sBP 140mmHg or dBP 90mmHg**				

**Pooled**	**Normal + Elevated BP + Stage 1 HTN (<140/90mmHg)**	**3.5 [2.5, 4.9] I^2^=0%,(2.3, 5.3)**	**0.93 [0.86, 0.97]**	**3.33 [2.23, 4.80]**

Bellad		2.9 [1.3, 6.7]	0.99 [0.98, 1.00]	3.36 [1.39, 8.09]
McLaren		1.3 [0.2, 10.6]	0.91 [0.62, 1.34]	1.58 [0.37, 6.76]
Qureshi		4.3 [2.7, 6.6]	0.96 [0.94, 0.99]	5.17 [3.00, 8.90]
Reddy		1.9 [0.5, 7.7]	0.98 [0.94, 1.02]	2.33 [0.68, 7.96]
Rosner		3.3 [0.2, 66.9]	0.88 [0.53, 1.47]	2.91 [0.22, 37.83]
Sabol		2.8 [0.9, 8.8]	0.82 [0.62, 1.09]	2.54 [1.14, 5.64]
Sevene		2.5 [0.4, 17.7]	0.95 [0.85, 1.06]	3.58 [0.70, 18.28]
Sutton 2020		2.8 [0.3, 23.6]	0.86 [0.62, 1.20]	3.39 [0.80, 14.36]

ACC (American College of Cardiology), AHA (American Heart Association), BP (blood pressure), CI (confidence interval), PI (prediction interval), dBP (diastolic BP), -LR (negative likelihood ratio), +LR (positive likelihood rate, sBP (systolic BP)

*Slade. The diagnostic accuracy of American College of Cardiology-American Heart Association blood pressure categories for identification of women and babies at risk. Am J Obstet Gynecol 2023*.

**Table S6:** Sensitivity and Specificity of ACC/AHA BP categories for serious maternal complications

		COMPARATOR	
BP measured at <20 weeks	Ref	Sensitivity	Specificity
**‘ELEVATED BP’ (as a new cut-off)**			
**Pooled**	**Normal (<120/80mmHg)**	**0.19 [0.12, 0.31]**	**0.84 [0.73, 0.91]**
Bellad		0.08 [0.05, 0.11]	0.95 [0.95, 0.95]
Qureshi		0.22 [0.18, 0.27]	0.86 [0.85, 0.87]
Reddy		0.21 [0.13, 0.31]	0.74 [0.73, 0.74]
Sevene		0.07 [0.02, 0.24]	0.89 [0.86, 0.91]
Sutton 2020		0.44 [0.17, 0.74]	0.75 [0.74, 0.76]
Rosner		0.17 [0.02, 0.69]	0.69 [0.64, 0.73]
**‘STAGE 1 HYPERTENSION’ (as a new cut-off)**			
**Pooled**	**Normal + Elevated BP (<130/80mmHg)**	**0.19 [0.11, 0.32]**	**0.87 [0.76, 0.94]**
Bellad		0.05 [0.03, 0.08]	0.97 [0.97, 0.98]
Duffy		0.31 [0.28, 0.33]	0.77 [0.77, 0.77]
McLaren		0.83 [0.44, 0.97]	0.51 [0.45, 0.57]
Qureshi		0.15 [0.11, 0.19]	0.92 [0.91, 0.92]
Reddy		0.10 [0.05, 0.19]	0.86 [0.86, 0.87]
Sabol		0.44 [0.23, 0.67]	0.79 [0.78, 0.81]
Sevene		0.05 [0.01, 0.22]	0.94 [0.92, 0.96]
Sutton 2020		0.14 [0.03, 0.51]	0.89 [0.89, 0.89]
**‘STAGE 2 HYPERTENSION’ (existing cut-off)**			
**Pooled**	**Normal + Elevated BP + Stage 1 HTN (<140/90mmHg)**	**0.10 [0.04, 0.20]**	**0.97 [0.93, 0.99]**
Bellad		0.02 [0.01, 0.03]	1.00 [0.99, 1.00]
McLaren		0.21 [0.05, 0.58]	0.86 [0.82, 0.90]
Qureshi		0.04 [0.03, 0.07]	0.99 [0.99, 0.99]
Reddy		0.03 [0.01, 0.10]	0.99 [0.98, 0.99]
Rosner		0.17 [0.02, 0.69]	0.94 [0.91, 0.96]
Sabol		0.26 [0.11, 0.50]	0.90 [0.86, 0.91]
Sevene		0.07 [0.02, 0.24]	0.98 [0.97, 0.99]
Sutton 2020		0.19 [0.03, 0.53]	0.94 [0.94, 0.95]

*Slade. The diagnostic accuracy of American College of Cardiology-American Heart Association blood pressure categories for identification of women and babies at risk. Am J Obstet Gynecol 2023*.

**Table S7:** Pooled relative risk and mean likelihood ratios for preterm birth, according to BP cut-offs at <20 weeks, determined by ACC/AHA BP categories

		COMPARATOR		
BP measured at <20 weeks	Ref	RR [95% CI] I^2^, (95% PI)	-LR	+LR
**Diagnostic threshold: sBP 120mmHg (with dBP <80mmHg)**				
**Pooled**	**Normal (<120/80mmHg)**	**1.2 [1.0, 1.5] I^2^=94%,** **(0.6, 2.5)**	**0.95 [0.89, 1.00]**	**1.21 [1.01, 1.43]**
Bellad		1.2 [1.0, 1.5]	0.99 [0.97, 1.00]	1.28 [1.03, 1.60]
Greenberg		1.6 [1.4, 1.8]	0.84 [0.80, 0.88]	1.41 [1.31, 1.52]
Hu		0.8 [0.7, 0.9]	1.09[ 1.03, 1.15]	0.86 [0.78, 0.96]
Qureshi		1.0 [0.9, 1.0]	1.01 [0.99, 1.03]	0.94 [0.83, 1.06]
Reddy		1.3 [1.1, 1.4]	0.93 [0.89, 0.97]	1.21 [1.10, 1.32]
Rosner		1.6 [0.9, 2.7]	0.83 [0.65, 1.07]	1.41 [0.96, 2.07]
Sevene		1.1 [0.8, 1.5]	0.98 [0.93, 1.04]	1.14 [0.74, 1.74]
Sutton 2020		1.8 [1.6, 2.0]	0.83 [0.79, 0.87]	1.54 [1.42, 1.67]
**Diagnostic threshold: sBP 130mmHg or dBP 80mmHg**				
**Pooled LR**	**Normal + ElevatedBP (<130/80mmHg)**	**1.5 [1.3, 1.8] I^2^=98%,** **(0.7, 3.3)**	**0.91 [0.84, 0.96]**	**1.49 [1.24, 1.77]**
Bellad		1.4 [1.1, 1.8]	0.99 [0.98, 1.00]	1.49 [1.11, 2.00]
Bello		2.0 [1.9, 2.1]	0.85 [0.84, 0.86]	1.83 [1.77, 1.90]
Darwin		2.3 [1.9, 2.8]	0.77 [0.71, 0.84]	2.04 [1.74, 2.40]
Duffy		1.2 [1.2, 1.3]	0.95 [0.94, 0.95]	1.18 [1.15, 1.21]
Greenberg		1.9 [1.7, 2.2]	0.88 [0.85, 0.91]	1.78 [1.59, 1.98]
Hu		0.8 [0.7, 1.0]	1.05 [1.01, 1.09]	0.86 [0.75, 0.99]
Huai		1.0 [0.6, 1.7]	1.01 [0.82, 1.24]	0.98 [0.66, 1.47]
McLaren		2.4 [1.2, 5.1]	0.55 [0.31, 0.96]	1.51 [1.16, 1.97]
Qureshi		1.0 [0.9, 1.2]	1.00 [0.98, 1.01]	1.04 [0.89, 1.22]
Reddy		1.4 [1.2, 1.7]	0.94 [0.91, 0.97]	1.38 [1.21, 1.58]
Sabol		2.2 [1.8, 2.6]	0.78 [0.72, 0.84]	1.99 [1.72, 2.30]
Sevene		1.1 [0.8, 1.7]	0.99 [0.95, 1.03]	1.21 [0.66, 2.22]
Sutton 2020		2.3 [2.0, 2.6]	0.87 [0.84, 0.90]	2.12 [1.88, 2.40]
**Diagnostic threshold: sBP 140mmHg or dBP 90mmHg**				
**Pooled LR**	**Normal + Elevated BP +Stage 1 (<140/90mmHg)**	**2.3 [2.0, 2.6] I^2^=82%,** **[1.1, 4.6]**	**0.95 [0.89, 0.98]**	**2.52 [2.04, 3.06]**
Bello		2.6 [2.5, 2.8]	0.92 [0.91, 0.93]	2.77 [2.60, 2.94]
Bellad		2.2 [1.4, 3.3]	0.99 [0.99, 1.00]	2.67 [1.46, 4.89]
Darwin		2.7 [2.2, 3.3]	0.82 [0.77, 0.87]	2.71 [2.20, 3.35]
Greenberg		2.5 [2.1, 2.9]	0.90 [0.88, 0.93]	2.46 [2.12, 2.85]
Hu		1.0 [0.7, 1.5]	1.00 [0.98, 1.01]	1.04 [0.73, 1.48]
McLaren		4.0 [2.2, 7.6]	0.68 [0.51, 0.90]	3.89 [2.15, 7.05]
Qureshi		1.6 [1.2, 2.0]	0.99 [0.99, 1.00]	2.14 [1.34, 3.43]
Reddy		2.4 [1.8, 3.3]	0.98 [0.97, 0.99]	2.66 [1.85, 3.82]
Rosner		2.1 [0.9, 4.7]	0.94 [0.84, 1.04]	2.25 [0.86, 5.85]
Sabol		2.7 [2.2, 3.2]	0.83 [0.79, 0.87]	2.93 [2.37, 3.62]
Sevene		1.3 [0.7, 2.4]	0.99 [0.97, 1.01]	1.48 [0.52, 4.21]
Sutton 2020		3.2 [2.7, 3.7]	0.89 [0.86, 0.91]	3.19 [2.74, 3.72]

ACC (American College of Cardiology), AHA (American Heart Association), BP (blood pressure), dBP (diastolic BP), -LR (negative likelihood ratio), +LR (positive likelihood ratio), sBP (systolic BP), CI (confidence interval), PI (prediction interval)

*Slade. The diagnostic accuracy of American College of Cardiology-American Heart Association blood pressure categories for identification of women and babies at risk. Am J Obstet Gynecol 2023*.

**Table S8:** Sensitivity and Specificity of ACC/AHA BP categories for preterm birth

		COMPARATOR	
BP measured at <20 weeks	Ref	Sensitivity	Specificity
**‘ELEVATED BP’ (as a new cut-off)**			
**Pooled**	**Normal (<120/80mmHg)**	**0.24 [0.14, 0.36]**	**0.80 [0.70, 0.88]**
Bellad		0.06 [0.05, 0.08]	0.95 [0.95, 0.96]
Greenberg		0.39 [0.36, 0.42]	0.72 [0.72, 0.73]
Hu		0.34 [0.30, 0.37]	0.61 [0.60, 0.62]
Qureshi		0.14 [0.13, 0.16]	0.85 [0.84, 0.86]
Reddy		0.31 [0.29, 0.34]	0.74 [0.73, 0.75]
Sevene		0.12 [0.08, 0.16]	0.90 [0.87, 0.92]
Sutton 2020		0.37 [0.34, 0.40]	0.76 [0.75, 0.77]
Rosner		0.41 [0.28, 0.56]	0.71 [0.66, 0.75]
**‘STAGE 1 HYPERTENSION’ (as a new cut-off)**			
**Pooled**	**Normal + Elevated BP (<130/80mmHg)**	**0.22 [0.14, 0.33]**	**0.85 [0.78, 0.90]**
Bellad		0.04 [0.03, 0.05]	0.97 [0.97, 0.98]
Bello		0.28 [0.28, 0.29]	0.84 [0.84, 0.85]
Darwin		0.37 [0.32, 0.42]	0.82 [0.81, 0.83]
Duffy		0.27 [0.27, 0.28]	0.77 [0.77, 0.77]
Greenberg		0.24 [0.22, 0.27]	0.86 [0.86, 0.87]
Hu		0.22 [0.19, 0.35]	0.75 [0.74, 0.75]
Huai		0.34 [0.23, 0.47]	0.65 [0.60, 0.70]
McLaren		0.71 [0.53, 0.84]	0.53 [0.46, 0.59]
Qureshi		0.09 [0.08, 0.10]	0.91 [0.90, 0.92]
Reddy		0.18 [0.16, 0.21]	0.87 [0.86, 0.87]
Sabol		0.36 [0.32, 0.41]	0.82 [0.80, 0.83]
Sevene		0.06 [0.04, 0.10]	0.95 [0.93, 0.95]
Sutton 2020		0.22 [0.19, 0.24]	0.90 [0.89, 0.90]
**‘STAGE 2 HYPERTENSION’ (existing cut-off)**			
**Pooled**	**Normal + Elevated BP +Stage 1 HTN (<140/90mmHg)**	**0.08 [0.04, 0.16]**	**0.97 [0.94, 0.98]**
Bellad		0.01 [0.01, 0.02]	1.00 [0.99, 1.00]
Bello		0.12 [0.11, 0.13]	0.96 [0.96, 0.96]
Darwin		0.26 [0.22, 0.31]	0.90 [0.89, 0.91]
Greenberg		0.15 [0.13, 0.18]	0.94 [0.93, 0.94]
Hu		0.04 [0.03, 0.06]	0.96 [0.96, 0.96]
McLaren		0.39 [0.24, 0.56]	0.90 [0.85, 0.93]
Qureshi		0.02 [0.01, 0.02]	0.99 [0.99, 0.99]
Reddy		0.03 [0.02, 0.04]	0.99 [0.99, 0.99]
Rosner		0.11 [0.05, 0.23]	0.95 [0.92, 0.97]
Sabol		0.24 [0.20, 0.28]	0.92 [0.91, 0.93]
Sevene		0.02 [0.01, 0.05]	0.98 [0.97, 0.99]
Sutton 2020		0.16 [0.14, 0.18]	0.95 [0.95, 0.95]

*Slade. The diagnostic accuracy of American College of Cardiology-American Heart Association blood pressure categories for identification of women and babies at risk. Am J Obstet Gynecol 2023*.

**Table S9:** Pooled relative risk and mean likelihood ratios for perinatal death, according to BP cut-offs at <20 weeks, determined by ACC/AHA BP categories

		COMPARATOR		
BP measured at <20 weeks	Ref	RR [95% CI] I^2^, (95% PI)	-LR	+LR
**Diagnostic threshold: sBP 120mmHg (with dBP <80mmHg)**				
**Pooled**	**Normal (<120/80 mmHg)**	**1.4 [1.2, 1.6]** **I^2^=11%, (1.1, 1.7)**	**0.94 [0.90, 0.97]**	**1.34 [1.17, 1.54]**
Bellad		1.4 [1.1, 1.9]	0.98 [0.95, 1.00]	1.44 [1.06, 1.95]
Greenberg		1.7 [1.1, 2.5]	0.84 [0.72, 0.99]	1.40 [1.09, 1.80]
Qureshi		1.4 [1.2, 1.7]	0.93 [0.90, 0.97]	1.40 [1.20, 1.65]
Reddy		0.9 [0.6, 1.4]	1.02 [0.91, 1.14]	0.94 [0.67, 1.31]
Sevene		1.1 [0.5, 2.6]	0.98 [0.88, 1.10]	1.12 [0.52, 2.43]
**Diagnostic threshold: sBP 130mmHg or dBP 80mmHg**				
**Pooled**	**Normal + Elevated BP (<130/80 mmHg)**	**1.6 [1.4, 1.9]** **I^2^=0%, (1.2, 2.1)**	**0.95 [0.92, 0.97]**	**1.59 [1.29, 1.94]**
Bellad		1.9 {1.4, 2.7]	0.97 [0.96, 0.99]	1.97 [1.36, 2.86]
Greenberg		1.8 [1.1, 2.9]	0.90 [0.80, 1.00]	1.62 [1.12, 2.35]
Qureshi		1.6 [1.3, 1.9]	0.95 [0.92, 0.98]	1.58 [1.28, 1.94]
Reddy		1.1 [0.6, 1.8]	0.99 [0.92, 1.07]	1.05 [0.65, 1.68]
Sevene		1.5 [0.5, 3.9]	0.97 [0.89, 1.06]	1.47 [0.55, 3.90]
**Diagnostic threshold: sBP 140mmHg or dBP 90mmHg**				
**Pooled**	**Normal + Elevated BP +Stage 1 HTN (<140/90 mmHg)**	**2.4 [1.8, 3.1]** **I^2^=0%, (1.5, 3.8)**	**0.98 [0.95, 0.99]**	**2.54 [1.76, 3.56]**
Bellad		2.1 [1.2, 3.8]	0.99 [0.98, 1.00]	3.60 [1.75, 7.38]
Greenberg		3.2 [1.7, 5.7]	0.93 [0.86, 1.00]	2.00 [1.20, 3.32]
Qureshi		2.3 [1.5, 3.5]	0.98 [0.97, 1.00]	2.59 [1.52, 4.43]
Reddy		1.4 [0.3, 5.7]	0.99 [0.97, 1.02]	1.40 [0.35, 5.58]
Sevene		2.3 [0.6, 8.6]	0.97 [0.92, 1.04]	2.41 [0.56, 10.30]

ACC (American College of Cardiology), AHA (American Heart Association), BP (blood pressure), dBP (diastolic BP), -LR (negative likelihood ratio), +LR (positive likelihood ratio), sBP (systolic BP), CI (confidence interval), PI (prediction interval)

*Slade. The diagnostic accuracy of American College of Cardiology-American Heart Association blood pressure categories for identification of women and babies at risk. Am J Obstet Gynecol 2023*.

**Table S10:** Sensitivity and Specificity of ACC/AHA BP categories for perinatal death

		COMPARATOR	
BP measured at <20 weeks	Ref	Sensitivity	Specificity
**‘ELEVATED BP’ (as a new cut-off)**			
**Pooled**	**Normal (<120/80mmHg)**	**0.20 [0.11, 0.33]**	**0.85 [0.74, 0.92]**
Bellad		0.07 [0.05, 0.10]	0.95 [0.95, 0.95]
Greenberg		0.40 [0.30, 0.50]	0.72 [0.71, 0.72]
Qureshi		0.20 [0.17, 0.23]	0.86 [0.85, 0.87]
Reddy		0.25 [0.17, 0.34]	0.74 [0.73, 0.74]
Sevene		0.13 [0.06, 0.25]	0.89 [0.86, 0.91]
**‘STAGE 1 HYPERTENSION’ (as a new cut-off)**			
**Pooled**	**Normal + Elevated BP (<130/80mmHg)**	**0.12 [0.07, 0.20]**	**0.92 [0.86, 0.96]**
Bellad		0.05 [0.04, 0.07]	0.97 [0.97, 0.98]
Greenberg		0.23 [0.16, 0.32]	0.86 [0.85, 0.86]
Qureshi		0.13 [0.11, 0.16]	0.92 [0.91, 0.92]
Reddy		0.14 [0.09, 0.22]	0.86 [0.86, 0.87]
Sutton 2020		0.09 [0.03, 0.20]	0.94 [0.92, 0.96]
**‘STAGE 2 HYPERTENSION’ (existing cut-off)**			
**Pooled**	**Normal + Elevated BP + Stage 1 HTN (<140/90mmHg)**	**0.04 [0.02, 0.08]**	**0.99 [0.96, 0.99]**
Bellad		0.01 [0.01, 0.02]	1.00 [0.99, 1.00]
Greenberg		0.14 [0.08, 0.22]	0.93 [0.93, 0.94]
Qureshi		0.02 [0.02, 0.04]	0.99 [0.99, 0.99]
Reddy		0.02 [0.01, 0.07]	0.99 [0.98, 0.99]
Sevene		0.04 [0.01, 0.14]	0.98 [0.97, 0.99]

*Slade. The diagnostic accuracy of American College of Cardiology-American Heart Association blood pressure categories for identification of women and babies at risk. Am J Obstet Gynecol 2023*.

**Table S11:** Pooled relative risk and mean likelihood ratios for stillbirth, according to BP cut-offs at <20 weeks, determined by ACC/AHA BP categories

		COMPARATOR		
BP measured at <20 weeks	Ref	RR [95% CI] I^2^, (95% PI)	-LR	+LR
**Diagnostic threshold: sBP 120mmHg (with dBP <80mmHg)**				
**Pooled**	**Normal (<120/80mmHg)**	**1.3 [0.9, 1.8] I^2^=51%, (0.4, 3.7)**	**0.97 [0.89, 1.04]**	**1.19 [0.86, 1.62]**
Bellad		1.5 [1.0, 2.2]	0.97 [0.95, 1.00]	1.49 [1.02, 2.17]
Qureshi		1.6 [1.3, 2.1]	0.91 [0.86, 0.96]	1.55 [1.27, 1.89]
Reddy		0.7 [0.4, 1.3]	1.07 [0.96, 1.20]	0.79 [0.52, 1.22]
Rosner		0.4 [0.0, 9.3]	1.21 [0.72, 2.01]	0.54 [0.04, 6.79]
Sevene		0.4 [0.1, 2.8]	1.05 [0.94, 1.18]	0.57 [0.12, 2.73]
Sutton 2020		2.3 [0.5, 10.1]	0.75 [0.41, 1.38]	1.76 [0.80, 3.86]
**Diagnostic threshold: sBP 130mmHg or dBP 80mmHg**				
**Pooled**	**Normal + Elevated BP(<130/80mmHg)**	**1.6 [1.3, 1.9] I^2^=29%, (1.1, 2.2)**	**0.93 [0.89, 0.96]**	**1.47 [1.28, 1.69]**
Bellad		1.8 [1.1, 2.9]	0.98 [0.95, 1.00]	1.81 [1.12, 2.92]
Duffy		1.5 [1.3, 1.6]	0.90 [0.87, 0.94]	1.31 [1.21, 1.43]
McLaren		5.0 [0.6, 42.2]	0.33 [0.05, 1.97]	1.69 [1.16, 2.48]
Qureshi		1.8 [1.4, 2.4]	0.93 [0.89, 0.97]	1.75 [1.36, 2.27]
Reddy		0.7 [0.3, 1.5]	1.04 [0.96, 1.12]	0.75 [0.39, 1.45]
Sabol		2.9 [1.1, 7.8]	0.71 [0.46, 1.10]	2.10 [1.20, 3.67]
Sevene		0.8 [0.1, 5.7]	1.01 [0.92, 1.11]	0.79 [0.11, 5.45]
Sutton 2020		1.4 [0.2, 11.2]	0.96 [0.71, 1.30]	1.30 [0.21, 8.00]
**Diagnostic threshold: sBP 140mmHg or dBP 90mmHg**				
**Pooled**	**Normal + Elevated BP** **+ Stage 1 HTN (<140/90mmHg)**	**3.5 [2.5, 4.0] I^2^=0%, (2.3, 5.3)**	**0.93 [0.86, 0.97]**	**3.33 [2.23, 4.80]**
Bellad		2.9 [1.3, 6.7]	0.99 [0.98, 1.00]	3.36 [1.39, 8.09]
McLaren		1.3 [0.2, 10.6]	0.91 [0.62, 1.34]	1.58 [0.37, 6.76]
Qureshi		4.3 [2.7, 6.6]	0.96 [0.94, 0.99]	5.17 [3.00, 8.90]
Reddy		1.9 [0.5, 7.7]	0.98 [0.94, 1.02]	2.33 [0.68, 7.96]
Rosner		3.3 [0.2, 66.9]	0.88 [0.53, 1.47]	2.91 [0.22, 37.83]
Sabol		2.8 [0.9, 8.8]	0.82 [0.62, 1.09]	2.54 [1.14, 5.64]
Sevene		2.5 [0.4, 17.7]	0.95 [0.85, 1.06]	3.58 [0.70, 18.28]
Sutton 2020		2.8 [0.3, 23.6]	0.86 [0.62, 1.20]	3.39 [0.80, 14.36]

ACC (American College of Cardiology), AHA (American Heart Association), BP (blood pressure), dBP (diastolic BP), -LR (negative likelihood ratio), +LR (positive likelihood ratio), sBP (systolic BP), CI (confidence interval), PI (prediction interval)

*Slade. The diagnostic accuracy of American College of Cardiology-American Heart Association blood pressure categories for identification of women and babies at risk. Am J Obstet Gynecol 2023*.

**Table S12:** Sensitivity and Specificity of ACC/AHA BP categories for stillbirth

		COMPARATOR	
BP measured at <20 weeks	Ref	Sensitivity	Specificity
**‘ELEVATED BP’ (as a new cut-off)**			
**Pooled**	**Normal (<120/80mmHg)**	**0.19 [0.12, 0.31]**	**0.84 [0.73, 0.91]**
Bellad		0.08 [0.05, 0.11]	0.95 [0.95, 0.95]
Qureshi		0.22 [0.18, 0.27]	0.86 [0.85, 0.87]
Reddy		0.21 [0.13, 0.31]	0.74 [0.73, 0.74]
Rosner		0.17 [0.02, 0.69]	0.69 [0.64, 0.73]
Sevene		0.07 [0.02, 0.24]	0.89 [0.86, 0.91]
Sutton 2020		0.44 [0.17, 0.74]	0.69 [0.64, 0.73]
**‘STAGE 1 HYPERTENSION’ (as a new cut-off)**			
**Pooled**	**Normal + Elevated BP (<130/80mmHg)**	**0.19 [0.11, 0.32]**	**0.87 [0.76, 0.94]**
Bellad		0.05 [0.03, 0.08]	0.97 [0.97, 0.98]
Duffy		0.31 [0.28, 0.33]	0.77 [0.77, 0.77]
McLaren		0.83 [0.44, 0.97]	0.51 [0.45, 0.57]
Qureshi		0.13 [0.11, 0.19]	0.92 [0.91, 0.92]
Reddy		0.10 [0.05, 0.19]	0.86 [0.86, 0.87]
Sabol		0.44 [0.23, 0.67]	0.79 [0.78, 0.81]
Sevene		0.05 [0.01, 0.22]	0.94 [0.92, 0.96]
Sutton 2020		0.14 [0.03, 0.51]	0.89 [0.89, 0.89]
**‘STAGE 2 HYPERTENSION’ (existing cut-off)**			
**Pooled**	**Normal + Elevated BP + Stage 1 HTN (<140/90mmHg)**	**0.10 [0.04, 0.20]**	**0.97 [0.93, 0.99]**
Bellad		0.02 [0.01, 0.03]	1.00 [0.99, 1.00]
McLaren		0.21 [0.05, 0.58]	0.86 [0.82, 0.90]
Qureshi		0.04 [0.03, 0.07]	0.99 [0.99, 0.99]
Reddy		0.03 [0.01, 0.10]	0.99 [0.98, 0.99]
Rosner		0.17 [0.02, 0.69]	0.94 [0.91, 0.96]
Sabol		0.26 [0.11, 0.50]	0.90 [0.88, 0.91]
Sevene		0.07 [0.02, 0.24]	0.98 [0.97, 0.99]
Sutton 2020		0.19 [0.04, 0.53]	0.94 [0.94, 0.95]

*Slade. The diagnostic accuracy of American College of Cardiology-American Heart Association blood pressure categories for identification of women and babies at risk. Am J Obstet Gynecol 2023*.

**Table S13:** Pooled relative risk and mean likelihood ratios for newborn death, according to BP cut-offs at <20 weeks, determined by ACC/AHA BP categories

		COMPARATOR		
BP measured at <20 weeks	Ref	RR [95% CI] I^2^, (95% PI)	-LR	+LR
**Diagnostic threshold: sBP 120mmHg (with dBP <80mmHg)**				
**Pooled**	**Normal (<120/80 mmHg)**	**1.4 [1.2, 1.7], I^2^=0% (1.0, 1.9)**	**0.94 [0.88, 0.98]**	**1.35 [1.14, 1.59]**
Bellad		1.4 [0.9, 2.2]	0.98 [0.95, 1.01]	1.44 [0.94, 2.20]
Qureshi		1.4 [1.1, 1.7]	0.95 [0.90, 0.99]	1.33 [1.07, 1.64]
Reddy		1.6 [0.8, 3.6]	0.85 [0.64, 1.13]	1.43 [0.88, 2.30]
Sevene		2.0 [0.9, 4.4]	0.89 [0.74, 1.06]	1.88 [0.97, 3.65]
Sutton 2020		1.3 [0.8, 2.2]	0.93 [0.79, 1.09]	1.22 [0.85, 1.77]
**Diagnostic threshold: sBP 130mmHg or dBP 80mmHg**				
**Pooled**	**Normal + Elevated BP(<130/80mmHg)**	**1.6 [1.3, 2.1] I^2^=15%, (1.0, 2.7)**	**0.96 [0.93, 0.98]**	**1.57 [1.19, 2.05]**
Bellad		2.2 [1.2, 3.5]	0.97 [0.94, 1.00]	2.17 [1.33, 3.55]
Qureshi		1.5 [1.1, 2.0]	0.96 [0.92, 0.99]	1.47 [1.12, 1.93]
Reddy		2.2 [0.9, 5.2]	0.86 [0.69, 1.07]	1.90 [1.00, 3.60]
Sevene		2.1 [0.8, 5.7]	0.94 [0.83, 1.06]	2.03 [0.78, 5.32]
Sutton 2020		0.9 [0.4, 2.0]	1.02 [0.94, 1.10]	0.87 [0.40, 1.86]
**Diagnostic threshold: sBP 140mmHg or dBP 90mmHg**				
**Pooled**	**Normal + Elevated BP** **+ Stage 1 HTN (<140/90mmHg)**	**1.9 [1.1, 3.4] I^2^=22%, (0.4, 9.4)**	**0.99 [0.99, 1.00]**	**2.04 [1.00, 3.73]**
Bellad		3.9 [1.7, 8.9]	0.98 [0.97, 1.00]	4.54 [1.87, 10.98]
Qureshi		1.8 [0.9, 3.7]	0.99 [0.98, 1.00]	2.00 [0.94, 4.25]
Reddy		1.3 [0.1, 21.5]	1.00 [0.95, 1.05]	1.31 [0.08, 20.46]
Sevene		1.6 [0.2, 11.2]	0.97 [0.91, 1.05]	2.35 [0.45, 12.19]
Sutton 2020		0.9 [0.3, 2.7]	1.00 [0.94, 1.06]	0.99 [0.36, 2.74]

ACC (American College of Cardiology), AHA (American Heart Association), BP (blood pressure), dBP (diastolic BP), -LR (negative likelihood ratio), +LR (positive likelihood ratio), sBP (systolic BP), CI (confidence interval), PI (prediction interval)

*Slade. The diagnostic accuracy of American College of Cardiology-American Heart Association blood pressure categories for identification of women and babies at risk. Am J Obstet Gynecol 2023*.

**Table S14:** Sensitivity and Specificity of ACC/AHA BP categories for newborn death

		COMPARATOR	
BP measured at <20 weeks	Ref	Sensitivity	Specificity
**‘ELEVATED BP’ (as a new cut-off)**			
**Pooled**	**Normal (<120/80 mmHg)**	**0.19 [0.11, 0.33]**	**0.89 [0.75, 0.92]**
Bellad		0.07 [0.05, 0.11]	0.95 [0.95, 0.95]
Qureshi		0.19 [0.15, 0.23]	0.86 [0.85, 0.87]
Reddy		0.37 [0.22, 0.56]	0.74 [0.73, 0.74]
Sevene		0.21 [0.10, 0.37]	0.89 [0.86, 0.91]
Sutton 2020		0.30 [0.20, 0.42]	0.75 [0.74, 0.76]
**‘STAGE 1 HYPERTENSION’ (as a new cut-off)**			
**Pooled**	**Normal + Elevated BP (<130/80mmHg)**	**0.11 [0.07, 0.17]**	**0.93 [0.87, 0.96]**
Bellad		0.06 [0.03, 0.09]	0.97 [0.97, 0.98]
Qureshi		0.12 [0.09, 0.16]	0.92 [0.91, 0.92]
Reddy		0.26 [0.13, 0.45]	0.86 [0.86, 0.87]
Sevene		0.12 [0.05, 0.27]	0.94 [0.92, 0.96]
Sutton 2020		0.10 [0.04, 0.19]	0.89 [0.89, 0.89]
**‘STAGE 2 HYPERTENSION’ (existing cut-off)**			
**Pooled**	**Normal + Elevated BP +Stage 1 HTN (<140/90mmHg)**	**0.03 [0.02, 0.05]**	**0.99 [0.97, 0.99]**
Sutton 2020		0.05 [0.02, 0.14]	0.94 [0.94 , 0.95]
Reddy		0.02 [0.00, 0.15]	0.99 [0.98, 0.99]
Bellad		0.02 [0.01, 0.04]	1.00 [0.99, 1.00]
Qureshi		0.02 [0.01, 0.04]	0.99 [0.99, 0.99]
Sevene		0.04 [0.01, 0.17]	0.98 [0.97, 0.99]

*Slade. The diagnostic accuracy of American College of Cardiology-American Heart Association blood pressure categories for identification of women and babies at risk. Am J Obstet Gynecol 2023*.

**Table S15:** Pooled relative risk and mean likelihood ratios for SGA infants, according to BP cut-offs at <20 weeks, determined by ACC/AHA BP categories

BP measured at <20 weeks	Ref	RR [95% CI] I^2^, (95% PI)	-LR	+LR
**Diagnostic threshold: sBP 120mmHg (with dBP <80mmHg)**				
**Pooled**	**Normal (<120/80 mmHg)**	**1.0 [0.9, 1.1] I^2^=79%, (0.7, 1.5)**	**1.00 [0.96, 1.04]**	**1.00 [0.91, 1.09]**
Greenberg		0.9 [0.9, 1.0]	1.02 [1.00,1.05]	0.94 [0.88, 1.01]
Hu		1.1 [1.0, 1.2]	0.96 [0.92, 1.00]	1.06 [1.00, 1.13]
Reddy		0.8 [0.7, 0.9]	1.05 [1.02, 1.08]	0.87 [0.78, 0.96]
Rosner		1.4 [0.7, 2.7]	0.89 [0.68, 1.18]	1.24 [0.77, 2.00]
Sutton 2020		1.1 [1.0, 1.2]	0.97 [0.94, 1.00]	1.09 [1.00, 1.17]
**Diagnostic threshold: sBP 130mmHg or dBP 80mmHg**				
**Pooled**	**Normal + ElevatedBP (<130/80 mmHg)**	**1.1 [1.0, 1.2] I^2^=78%,** **[0.8, 1.4]**	**0.98 [0.96, 1.00]**	**1.06 [0.99, 1.14]**
Bello		1.2 [1.1, 1.2]	0.97 [0.96, 0.98]	1.15 [1.10, 1.20]
Darwin		1.4 [1.1, 1.7]	0.92 [0.86, 0.99]	1.31 [1.07, 1.61]
Duffy		1.0 [1.0, 1.1]	0.99 [0.99, 1.00]	1.02 [1.00, 1.04]
Greenberg		1.0 [0.9, 1.2]	0.99 [0.98, 1.01]	1.04 [0.93, 1.15]
Hu		1.1 [1.0, 1.2]	0.97 [0.95, 1.00]	1.08 [0.99, 1.17]
Huai		0.9 [0.5, 1.7]	1.03 [0.82, 1.30]	0.94 [0.59, 1.50]
McLaren		1.2 [0.6, 2.6]	0.88 [0.56, 1.36]	1.13 [0.78, 1.62]
Reddy		0.9 [0.7, 1.0]	1.02 [1.00, 1.04]	0.87 [0.75, 1.01]
Sabol		0.9 [0.7, 1.1]	1.03 [0.98, 1.08]	0.90 [0.73, 1.11]
Sutton 2020		1.2 [1.1, 1.4]	0.97 [0.96, 0.99]	1.21 [1.07, 1.37]
**Diagnostic threshold: sBP 140mmHg or dBP 90mmHg**				
**Pooled**	**Normal + Elevated BP +Stage 1 HTN (<140/90 mmHg)**	**1.3 [1.2, 1.4] I^2^=47%, (1.0, 1.6)**	**0.98 [0.96, 0.99]**	**1.32 [1.17, 1.48]**
Bello		1.3 [1.2 ,1.4]	0.98 [0.98, 0.99]	1.35 [1.25, 1.46]
Darwin		1.2 [0.9, 1.7]	0.97 [0.92, 1.02]	1.24 [0.92, 1.66]
Greenberg		1.2 [1.0, 1.4]	0.98 [0.97, 1.00]	1.22 [1.05, 1.42]
Hu		1.3 [1.1, 1.6]	0.99 [0.98, 1.00]	1.31 [1.06, 1.62]
Reddy		0.7 [0.4, 1.2]	1.00 [1.00, 1.01]	0.72 [0.42, 1.24]
Rosner		2.4 [0.9, 6.3]	0.92 [0.81, 1.05]	2.54 [0.91, 7.09]
McLaren		1.8 [0.8, 4.2]	0.89 [0.72, 1.09]	1.79 [0.81, 3.92]
Sabol		1.1 [0.8, 1.5]	0.99 [0.95, 1.02]	1.12 [0.83, 1.50]
Sutton 2020		1.6 [1.3, 1.8]	0.97 [0.95, 0.98]	1.61 [1.37, 1.89]

ACC (American College of Cardiology), AHA (American Heart Association), BP (blood pressure), (diastolic BP), -LR (negative likelihood ratio), +LR (positive likelihood ratio), sBP (systolic BP), SGA (small-for-gestational age), CI (confidence interval), PI (prediction interval)

*Slade. The diagnostic accuracy of American College of Cardiology-American Heart Association blood pressure categories for identification of women and babies at risk. Am J Obstet Gynecol 2023*.

**Table S16:** Sensitivity and Specificity of ACC/AHA BP categories for SGA infants

		COMPARATOR	
BP measured at <20 weeks	Ref	Sensitivity	Specificity
**‘ELEVATED BP’ (as a new cut-off)**			
**Pooled**	**Normal (<120/80mmHg)**	**0.30 [0.24, 0.36]**	**0.70 [0.65, 0.75]**
Greenberg		0.27 [0.25, 0.29]	0.71 [0.71, 0.72]
Hu		0.41 [0.39, 0.43]	0.61 [0.61, 0.62]
Reddy		0.23 [0.21, 0.25]	0.73 [0.73, 0.74]
Rosner		0.38 [0.23, 0.55]	0.70 [0.65, 0.74]
Sutton		0.27 [0.25, 0.29]	0.75 [0.75, 0.76]
**‘STAGE 1 HYPERTENSION’ (as a new cut-off)**			
**Pooled**	**Normal + Elevated BP (<130/80mmHg)**	**0.22 [0.17, 0.30]**	**0.79 [0.72, 0.84]**
Bello		0.19 [0.18, 0.20]	0.84 [0.83, 0.84]
Darwin		0.26 [0.21, 0.31]	0.80 [0.79, 0.82]
Duffy		0.24 [0.23, 0.24]	0.77 [0.77, 0.77]
Greenberg		0.15 [0.13, 0.16]	0.86 [0.85, 0.86]
Hu		0.27 [0.25, 0.29]	0.75 [0.74, 0.76]
Huai		0.32 [0.20, 0.48]	0.65 [0.60, 0.70]
McLaren		0.56 [0.37, 0.72]	0.51 [0.44, 0.57]
Reddy		0.12 [0.10, 0.14]	0.86 [0.89, 0.90]
Sabol		0.19 [0.16, 0.23]	0.79 [0.77, 0.80]
Sutton 2020		0.13 [0.12, 0.15]	0.89 [0.89, 0.90]
**‘STAGE 2 HYPERTENSION’ (existing cut-off)**			
**Pooled**	**Normal + Elevated BP** **+ Stage 1 HTN (<140/90mmHg)**	**0.08 [0.05, 0.13]**	**0.94 [0.91, 0.96]**
Hu		0.05 [0.04, 0.06]	0.96 [0.96, 0.96]
Greenberg		0.08 [0.07, 0.09]	0.93 [0.93, 0.94]
Sutton		0.08 [0.07, 0.10]	0.95 [0.94, 0.95]
Reddy		0.01 [0.01, 0.02]	0.99 [0.98, 0.99]
McLaren		0.22 [0.11, 0.41]	0.88 [0.83, 0.91]
Darwin		0.14 [0.11, 0.18]	0.89 [0.87, 0.90]
Bello		0.06 [0.06, 0.07]	0.95 [0.95, 0.95]
Sabol		0.12 [0.09, 0.15]	0.90 [0.88, 0.91]
Rosner		0.12 [0.05, 0.28]	0.95 [0.92, 0.97]

*Slade. The diagnostic accuracy of American College of Cardiology-American Heart Association blood pressure categories for identification of women and babies at risk. Am J Obstet Gynecol 2023*.

**Table S17:** Pooled relative risk and mean likelihood ratios for NICU admission, according to BP cut-offs at <20 weeks, determined by ACC/AHA BP categories

		COMPARATOR		
BP measured at <20 weeks	Ref	RR [95% CI] I^2^, (95% PI)	-LR	+LR
**Diagnostic threshold: sBP 120mmHg (with dBP <80mmHg)**				
**Pooled**	**Normal (<120/80 mmHg)**	**1.5 [1.2, 1.8] I^2^=45%, (0.2, 10.15)**	**0.87 [0.82, 0.92]**	**1.36 [1.21, 1.52]**
Greenberg		1.3 [1.3, 1.4]	0.90 [0.87, 0.92]	1.27 [1.20, 1.35]
Reddy		1.6 [1.2, 2.1]	0.87 [0.78, 0.96	1.38 [1.15, 1.86]
Rosner		2.1 [1.2, 3.5]	0.73 [0.55, 0.96]	1.70 [1.20, 2.39]
**Diagnostic threshold: sBP 130mmHg or dBP 80mmHg**				
**Pooled**	**Normal + ElevatedBP (<130/80 mmHg)**	**1.4 [1.2, 1.7] I^2^=96%, (1.1, 1.8)**	**0.92 [0.86, 0.97]**	**1.39 [1.11, 1.72]**
Bello		1.7 [1.6, 1.7]	0.89 [0.88, 0.90]	1.56 [1.50, 1.62]
Darwin		2.2 [1.8, 2.7]	0.78 [0.72, 0.85]	1.97 [1.66, 2.32]
Duffy		1.2 [1.1, 1.3]	0.96 [0.94, 0.98]	1.14 [1.08, 1.19]
Greenberg		1.4 [1.3, 1.6]	0.93 [0.92, 0.95]	1.43 [1.31, 1.56]
Reddy		1.7 [1.2, 2.3]	0.91 [0.85, 0.98]	1.55 [1.18, 2.03]
Sabol		0.9 [0.8, 1.1]	1.03 [0.98, 1.07]	0.91 [0.76, 1.09]
**Diagnostic threshold: sBP 140mmHg or dBP 90mmHg**				
**Pooled**	**Normal + Elevated BP** **+ Stage 1 HTN (<140/90 mmHg)**	**1.8 [1.3, 2.4] I^2^=93%, (0.4, 9.0)**	**0.95 [0.91, 0.99]**	**1.98 [1.18, 3.15]**
Bello		2.0 [1.9, 2.1]	0.95 [0.94, 0.96]	2.06 [1.92, 2.20]
Darwin		2.4 [1.9, 3.0]	0.84 [0.79, 0.90]	2.40 [1.92, 2.99]
Greenberg		1.5 [1.4, 1.7]	0.96 [0.95, 0.97]	1.62 [1.43, 1.85]
Reddy		4.1 [2.3, 7.4]	0.96 [0.93, 0.99]	4.11 [2.28, 7.43]
Rosner		2.0 [0.9, 4.5]	0.94 [0.85, 1.04]	2.14 [0.82, 5.58]
Sabol		0.7 [0.6, 1.0]	1.04 [1.01, 1.07]	0.71 [0.53, 0.96]

ACC (American College of Cardiology), AHA (American Heart Association), BP (blood pressure), dBP (diastolic BP), -LR (negative likelihood ratio), +LR (positive likelihood ratio), NICU (neonatal intensive care unit), sBP (systolic BP), CI (confidence interval), PI (prediction interval)

*Slade. The diagnostic accuracy of American College of Cardiology-American Heart Association blood pressure categories for identification of women and babies at risk. Am J Obstet Gynecol 2023*.

**Table S18:** Sensitivity and Specificity of ACC/AHA BP categories for NICU admission

		COMPARATOR	
BP measured at <20 weeks	Ref	Sensitivity	Specificity
**‘ELEVATED BP’ (as a new cut-off)**			
**Pooled**	**Normal (<120/80 mmHg)**	**0.36 [0.33, 0.39]**	**0.73 [0.61, 0.74]**
Greenberg		0.35 [0.33, 0.37]	0.73 [0.72, 0.73]
Reddy		0.36 [0.30, 0.43]	0.74 [0.73, 0.75]
Rosner		0.48 [0.34, 0.62]	0.72 [0.67, 0.76]
**‘STAGE 1 HYPERTENSION’ (as a new cut-off)**			
**Pooled**	**Normal + Elevated BP (<130/80mmHg)**	**0.24 [0.20, 0.29]**	**0.83 [0.79, 0.86]**
Bello		0.25 [0.24, 0.26]	0.84 [0.84, 0.84]
Darwin		0.36 [0.31, 0.41]	0.82 [0.80, 0.83]
Duffy		0.27 [0.25, 0.28]	0.77 [0.77, 0.77]
Greenberg		0.19 [0.18, 0.21]	0.87 [0.86, 0.87]
Reddy		0.21 [0.16, 0.27]	0.86 [0.86, 0.87]
Sabol		0.19 [0.16, 0.23]	0.79 [0.77, 0.80]
**‘STAGE 2 HYPERTENSION’ (existing cut-off)**			
**Pooled**	**Normal + Elevated BP** **+ Stage 1 HTN (<140/90mmHg)**	**0.10 [0.06, 0.16]**	**0.95 [0.90, 0.97]**
Bello		0.09 [0.09, 0.10]	0.95 [0.95, 0.96]
Darwin		0.24 [0.20, 0.29]	0.90 [0.89, 0.91]
Greenberg		0.10 [0.09, 0.11]	0.94 [0.93, 0.94]
Reddy		0.05 [0.03, 0.09]	0.99 [0.99, 0.99]
Rosner		0.19 [0.05, 0.22]	0.95 [0.92, 0.97]
Sabol		0.08 [0.06, 0.10]	0.89 [0.88, 0.90]

*Slade. The diagnostic accuracy of American College of Cardiology-American Heart Association blood pressure categories for identification of women and babies at risk. Am J Obstet Gynecol 2023*.

**Table S19:** Pooled relative risk and mean likelihood ratios for eclampsia, according to BP cut-offs at <20 weeks, determined by ACC/AHA BP categories

		COMPARATOR		
BP measured at <20 weeks	Ref	RR [95% CI] I^2^, (95% PI)	-LR	+LR
**Diagnostic threshold: sBP 120mmHg (with dBP <80mmHg)**				
**Pooled**	**Normal (<120/80 mmHg)**	**2.2 [0.8, 6.5] I^2^=40%, (UTD)**	**0.87 [0.62, 1.00]**	**1.73 [0.97, 2.60]**
Bellad		0.5 [0.1, 3.7]	1.01 [0.95, 1.08]	0.76 [0.16, 3.64]
Greenberg		2.7 [1.3, 5.5]	0.68 [0.47, 0.97]	1.82 [1.30, 2.55]
Qureshi		5.7 [0.8, 40.6]	0.59 [0.24, 1.41]	3.36 [1.40,8.09]
Sevene		-	0.57 [0.08, 4.05]	4.07 [0.57, 29.11]
**Diagnostic threshold: sBP 130mmHg or dBP 80mmHg**				
**Pooled**	**Normal + Elevated BP(<130/80 mmHg)**	**2.5 [1.1, 5.4] I^2^=66%, (0.1, 48.6)**	**0.84 [0.67, 0.96]**	**2.57 [1.39, 4.32]**
Bellad		0.9 [0.1, 6.7]	0.99 [0.93, 1.06]	1.35 [0.28, 6.51]
Duffy		1.5 [1.2, 5.4]	0.89 [0.81, 0.97]	1.37 [1.13, 1.65]
Greenberg		3.8 [1.9, 7.9]	0.71 [0.54, 0.94]	2.76 [1.79, 4.27]
Qureshi		10.2 [1.4, 72.2]	0.55 [0.23, 1.32]	5.60 [2.32, 13.50]
Sevene		-	0.53 [0.08, 3.79]	7.75 [1.07, 55.88]
**Diagnostic threshold: sBP 140mmHg or dBP 90mmHg**				
**Pooled**	**Normal + Elevated BP** **+ Stage 1 HTN (<140/90 mmHg)**	**7.3 [3.8, 14.3] I^2^=0% (0.1, 560.9)**	**0.87 [0.69, 0.96]**	**7.79 [4.16, 13.30]**
Bellad		5.2 [0.7, 37.6]	0.96 [0.90, 1.03]	7.50 [1.53, 36.65]
Greenberg		7.5 [3.6, 15.7]	0.69 [0.53, 0.89]	5.31 [3.34, 8.64]
Qureshi		10.0 [0.5, 183.7]	0.91 [0.68, 1.22]	9.07 [0.65, 126.89]
Sevene		-	0.51 [0.07, 3.62]	24.08 [3.22, 179.93]

ACC (American College of Cardiology), AHA (American Heart Association), BP (blood pressure), dBP (diastolic BP), -LR- (negative likelihood ratio), +LR (positive likelihood ratio), sBP (systolic BP), UTD (unable to determine), CI (confidence interval), PI (prediction interval)

*Slade. The diagnostic accuracy of American College of Cardiology-American Heart Association blood pressure categories for identification of women and babies at risk. Am J Obstet Gynecol 2023*.

**Table S20:** Sensitivity and Specificity of ACC/AHA BP categories for eclampsia

		COMPARATOR	
BP measured at <20 weeks	Ref	Sensitivity	Specificity
**‘ELEVATED BP’ (as a new cut-off)**			
**Pooled**	**Normal (<120/80 mmHg)**	**0.23 [0.08, 0.52]**	**0.89 [0.75, 0.94]**
Bellad		0.04 [0.01, 0.16]	0.95 [0.94, 0.95]
Greenberg		0.52 [0.35, 0.68]	0.72 [0.71, 0.72]
Qureshi		0.50 [0.17, 0.83]	0.85 [0.84, 0.86]
Sevene		0.50 [0.05, 0.95]	0.88 [0.85, 0.90]
**‘STAGE 1 HYPERTENSION’ (as a new cut-off)**			
**Pooled**	**Normal + Elevated BP (<130/80mmHg)**	**0.23 [0.11, 0.42]**	**0.91 [0.82, 0.96]**
Bellad		0.25 [0.24, 0.26]	0.84 [0.84, 0.84]
Greenberg		0.39 [0.24, 0.56]	0.86 [0.85, 0.86]
Duffy		0.32 [0.26, 0.38]	0.77 [0.76, 0.77]
Qureshi		0.50 [0.17, 0.83]	0.91 [0.90, 0.92]
Sevene		0.50 [0.05, 0.95]	0.94 [0.92, 0.95]
**‘STAGE 2 HYPERTENSION’ (existing cut-off)**			
**Pooled**	**Normal + Elevated BP +Stage 1 HTN (<140/90mmHg)**	**0.13 [0.04, 0.34]**	**0.98 [0.95, 0.99]**
Bellad		0.04 [0.01, 0.16]	0.99 [0.99, 1.00]
Greenberg		0.36 [0.22, 0.53]	0.93 [0.93, 0.94]
Qureshi		0.10 [0.01, 0.54]	0.99 [0.99, 0.99]
Sevene		0.50 [0.05, 0.95]	0.98 [0.97, 0.99]

*Slade. The diagnostic accuracy of American College of Cardiology-American Heart Association blood pressure categories for identification of women and babies at risk. Am J Obstet Gynecol 2023*.

**Table S21:** Pooled relative risk and mean likelihood ratios for stroke, according to BP cut-offs at <20 weeks, determined by ACC/AHA BP categories

		COMPARATOR		
BP measured at <20 weeks	Ref	RR [95% CI] I^2^, (95% PI)	-LR	+LR
**Diagnostic threshold: sBP 120mmHg (with dBP <80mmHg)**				
**Pooled**	**Normal (<120/80 mmHg)**	**2.1 [0.7, 6.2], I^2^= 63% (UTD)**	**0.89 [0.65, 1.03]**	**2.08 [0.74, 4.39]**
Bellad		3.5 [1.0, 12.2]	0.88 [0.72, 1.08]	3.13[1.11, 8.81]
Qureshi		3.4 [1.5, 7.8]	0.73 [0.54, 1.00]	2.52 [1.50, 4.23]
Sevene		0.6 [0.1, 2.4]	1.05 [0.95, 1.17]	0.59 [0.15, 2.28]
**Diagnostic threshold: sBP 130mmHg or dBP 80mmHg**				
**Pooled**	**Normal + ElevatedBP (<130/80mmHg)**	**2.1 [0.8, 5.6] I^2^=76%, (0.0, 105.9)**	**0.86 [0.84, 0.91]**	**2.8 [1.4, 5.2]**
Bellad		6.5 [1.9, 22.2]	0.84 [0.68, 1.04]	6.18 [2.39, 16.01]
Duffy		1.3 [1.1, 1.4]	0.94 [0.91, 0.97]	1.19 [1.09, 1.29]
Qureshi		3.4 [1.4, 8.5]	0.81 [0.64, 1.02]	2.93 [1.50, 5.69]
Sevene		0.3 [0.0, 4.3]	1.05 [1.00, 1.10]	0.27 [0.02, 4.21]
**Diagnostic threshold: sBP 140mmHg or dBP 90mmHg**				
**Pooled**	**Normal + Elevated BP +Stage 1 HTN (<140/90 mmHg)**	**8.6 [1.5, 50.2] I^2^=70%, (UTD)**	**0.90 [0.77, 0.98]**	**12.7 [2.11, 41.10]**
Bellad		22.8 [5.4, 97.3]	0.87 [0.73, 1.04]	24.72 [7.58, 80.60]
Qureshi		12.6 [3.8, 41.4]	0.87 [0.74, 1.02]	12.87 [4.75, 34.88]
Sevene		0.9 [0.1, 13.5]	1.00 [0.96, 1.05]	0.83 [0.05, 13.53]

ACC (American College of Cardiology), AHA (American Heart Association), BP (blood pressure), dBP (diastolic BP), -LR- (negative likelihood ratio), +LR (positive likelihood ratio), sBP (systolic BP), UTD (unable to determine), CI (Confidence interval), PI (prediction interval)

*Slade. The diagnostic accuracy of American College of Cardiology-American Heart Association blood pressure categories for identification of women and babies at risk. Am J Obstet Gynecol 2023*.

**Table S22:** Sensitivity and Specificity of ACC/AHA BP categories for stroke

		COMPARATOR	
BP measured at <20 weeks	Ref	Sensitivity	Specificity
**‘ELEVATED BP’ (as a new cut-off)**			
**Pooled**	**Normal (<120/80 mmHg)**	**0.19 [0.06, 0.43]**	**0.90 [0.82, 0.95]**
Bellad		0.17 [0.06, 0.39]	0.95 [0.94, 0.95]
Qureshi		0.38 [0.21, 0.57]	0.85 [0.84, 0.86]
Sevene		0.07 [0.02, 0.22]	0.88 [0.86, 0.90]
**‘STAGE 1 HYPERTENSION’ (as a new cut-off)**			
**Pooled**	**Normal** **+ Elevated BP (<130/80mmHg)**	**0.21 [0.17, 0.27]**	**0.92 [0.82, 0.97]**
Bellad		0.25 [0.24, 0.26]	0.84 [0.84, 0.84]
Duffy		0.28 [0.26, 0.30]	0.77 [0.76, 0.77]
Qureshi		0.26 [0.13, 0.46]	0.91 [0.90, 0.92]
Sevene		0.02 [0.00, 0.14]	0.94 [0.92, 0.95]
**‘STAGE 2 HYPERTENSION’ (existing cut-off)**			
**Pooled**	**Normal + Elevated BP +Stage 1 HTN (<140/90mmHg)**	**0.10 [0.04, 0.24]**	**0.99 [0.98, 1.00]**
Bellad		0.13 [0.04, 0.35]	0.99 [0.99, 1.00]
Qureshi		0.14 [0.05, 0.32]	0.99 [0.99, 0.99]
Sevene		0.02 [0.00, 0.14]	0.98 [0.97, 0.99]

*Slade. The diagnostic accuracy of American College of Cardiology-American Heart Association blood pressure categories for identification of women and babies at risk. Am J Obstet Gynecol 2023*.

**Table S23:** Pooled relative risk and mean likelihood ratios for maternal ICU admission, according to BP cut-offs at <20 weeks, determined by ACC/AHA BP categories

		COMPARATOR		
BP measured at <20 weeks	Ref	RR [95%CI] I^2^, (95% PI)	-LR	+LR
**Diagnostic threshold: sBP 120mmHg (with dBP <80mmHg)**				
**Pooled**	**Normal (<120/80 mmHg)**	**1.5 [1.1, 2.0] I^2^= 0%, (0.8, 2.9)**	**0.86 [0.79, 0.92]**	**2.02 [1.31, 3.06]**
Bellad		2.5 [0.3, 20.5]	0.92 [0.71, 1.20]	2.34 [0.37, 14.66]
Greenberg		1.5 [1.1, 2.0]	0.89 [0.79, 0.99]	1.29 [1.06, 1.57]
Qureshi		3.8 [0.6, 22.6]	0.71 [0.34, 1.44]	2.67 [0.91, 7.83]
Sevene		2.6 [0.3, 24.4]	0.85 [0.48, 1.49]	2.81 [0.40, 12.04]
**Diagnostic threshold: sBP 130mmHg or dBP 80mmHg**				
**Pooled**	**Normal + ElevatedBP (<130/80mmHg)**	**2.3 [1.7, 3.1] I^2^= 0%, (1.1, 4.6)**	**0.94 [0.84, 0.99]**	**1.78 [1.28, 2.35]**
Bellad		1.9 [0.1, 32.9]	0.97 [0.83, 1.14]	1.85 [0.12, 27.40]
Greenberg		2.3 [1.7, 3.2]	0.84 [0.77, 0.92]	1.98 [1.56, 2.51]
Qureshi		0.9 [0.1, 16.7]	1.01 [0.79, 1.28]	0.93 [0.07, 13.25]
Sevene		1.7 [0.1, 31.6]	0.96 [0.72, 1.28]	1.65 [0.12, 23.14]
**Diagnostic threshold: sBP 140mmHg or dBP 90mmHg**				
**Pooled**	**Normal + Elevated BP** **+ Stage 1 HTN (<140/90 mmHg)**	**4.0 [2.8, 5.6] I^2^= 0%, (1.9, 8.4)**	**0.91 [0.84, 0.96]**	**6.34 [3.55, 10.50]**
Bellad		3.9 [2.7, 5.5]	0.83 [0.77, 0.90]	3.34 [2.53, 4.41]
Greenberg		10.7 [0.6, 182.8]	0.95 [0.81, 1.11]	10.12 [0.68, 151.30]
Qureshi		8.0 [0.4, 143.0]	0.93 [0.73, 1.18]	7.39 [0.52, 105.91]
Sevene		5.6 [0.3, 100.3]	0.92 [0.69, 1.23]	5.14 [0.35, 74.41]

ACC (American College of Cardiology), AHA (American Heart Association), BP (blood pressure), dBP (diastolic BP), -LR- (negative likelihood ratio), +LR (positive likelihood ratio), sBP (systolic BP), CI (confidence interval), PI (prediction interval)

*Slade. The diagnostic accuracy of American College of Cardiology-American Heart Association blood pressure categories for identification of women and babies at risk. Am J Obstet Gynecol 2023*.

**Table S24:** Sensitivity and Specificity of ACC/AHA BP categories for maternal ICU admission

		COMPARATOR	
BP measured at <20 weeks	Ref	Sensitivity	Specificity
**‘ELEVATED BP’ (as a new cut-off)**			
**Pooled**	**Normal (<120/80mmHg)**	**0.26 [0.17, 0.37]**	**0.87 [0.75, 0.94]**
Bellad		0.12 [0.02, 0.47]	0.95 [0.94, 0.95]
Greenberg		0.36 [0.30, 0.44]	0.72 [0.71, 0.72]
Qureshi		0.40 [0.12, 0.77]	0.85 [0.84, 0.86]
Sevene		0.25 [0.05, 0.70]	0.89 [0.86, 0.91]
**‘STAGE 1 HYPERTENSION’ (as a new cut-off)**			
**Pooled**	**Normal + Elevated BP (<130/80mmHg)**	**0.13 [0.05, 0.27]**	**0.93 [0.87, 0.96]**
Bellad		0.06 [0.01, 0.37]	0.97 [0.97, 0.97]
Greenberg		0.28 [0.22, 0.35]	0.86 [0.85, 0.86]
Qureshi		0.08 [0.01, 0.48]	0.91 [0.90, 0.92]
Sevene		0.10 [0.01, 0.54]	0.94 [0.92, 0.95]
**‘STAGE 2 HYPERTENSION’ (existing cut-off)**			
**Pooled**	**Normal + Elevated BP +Stage 1 HTN (<140/90mmHg)**	**0.10 [0.05, 0.20]**	**0.98 [0.95, 0.99]**
Bellad		0.06 [0.01, 0.37]	0.99 [0.99, 1.00]
Greenberg		0.22 [0.17, 0.29]	0.93 [0.93, 0.94]
Qureshi		0.08 [0.01, 0.48]	0.99 [0.99, 0.99]
Sevene		0.10 [0.01, 0.54]	0.98 [0.97, 0.99]

*Slade. The diagnostic accuracy of American College of Cardiology-American Heart Association blood pressure categories for identification of women and babies at risk. Am J Obstet Gynecol 2023*.

**Table S25:** Pooled relative risk and mean likelihood ratios for placental abruption, according to BP cut-offs at <20 weeks, determined by ACC/AHA BP categories

		COMPARATOR		
BP measured at <20 weeks	Ref	RR [95% CI] I^2^, (95% PI)	-LR	+LR
**Diagnostic threshold: sBP 120mmHg (with dBP <80mmHg)**				
**Pooled LR**	**Normal (<120/80 mmHg)**	**1.1 [0.9, 1.3] I^2^=0%, (0.7, 1.6)**	**0.98 [0.93, 1.03]**	**1.05 [0.90, 1.22]**
Greenberg		1.1 [0.8, 1.5]	0.98 [0.89, 1.08]	1.05 [0.84, 1.32]
Qureshi		1.0 [0.6, 1.7]	1.00 [0.90. 1.10]	1.02 [0.60, 1.74]
Sevene		0.8 [0.1, 10.1]	1.20 [0.40, 3.62]	0.67 [0.04, 10.05]
Sutton 2020		1.1 [0.8, 1.5]	0.97 [0.90, 1.05]	1.08 [0.88, 1.32]
**Diagnostic threshold: sBP 130mmHg or dBP 80mmHg**				
**Pooled LR**	**Normal + ElevatedBP (<130/80 mmHg)**	**1.1 [1.0, 1.2] I^2^=0%, (1.0, 1.3)**	**0.97 [0.94, 0.99]**	**1.10 [1.04, 1.17]**
Duffy		1.1 [1.1, 1.2]	0.97 [0.95, 0.99]	1.11 [1.04, 1.18]
Huai		1.0 [0.6, 1.9]	0.99 [0.78, 1.25]	1.03 [0.66, 1.59]
Greenberg		1.3 [0.9, 1.9]	0.96 [0.89, 1.02]	1.27 [0.92, 1.74]
McLaren		1.0 [0.1, 15.8]	1.00 [0.32, 3.12]	1.00 [0.32, 3.12]
Qureshi		1.2 [0.7, 2.1]	0.97 [0.89, 1.06]	1.28 [0.66, 2.47]
Sabol		1.0 [0.5, 2.1]	0.99 [0.85, 1.16]	1.03 [0.58, 1.81]
Sevene		0.8 [0.1, 10.1]	1.20 [0.40, 3.62]	0.67 [0.04, 10.05]
Sutton 2020		0.8 [0.6, 1.3]	1.02 [0.98, 1.06]	0.87 [0.60, 1.28]
**Diagnostic threshold: sBP 140mmHg or dBP 90mmHg**				
**Pooled LR**	**Normal + Elevated BP +Stage 1 HTN (<140/90 mmHg)**	**1.4 [1.0, 1.9] I^2^=0%, (0.8, 2.3)**	**0.97 [0.91, 1.00]**	**1.41 [1.04, 1.85]**
Greenberg		1.6 [1.0, 2.5]	0.96 [0.91, 1.01]	1.55 [1.00, 2.39]
Sutton 2020		1.1 [0.7, 1.9]	0.99 [0.96, 1.02]	1.17 [0.73, 1.87]
Qureshi		1.9 [0.6, 5.7]	0.98 [0.95, 1.02]	2.65 [0.53, 13.18]
Sevene		N/A	0.86 [0.35, 2.07]	2.00 [0.06, 68.48]
McLaren		6.4 [0.4, 100.1]	0.58 [0.19, 1.79]	3.75 [1.16, 12.13]
Sabol		1.1 [0.3, 2.8]	0.98 [0.87, 1.09]	1.19 [0.54, 2.62]

ACC (American College of Cardiology), AHA (American Heart Association), BP (blood pressure), dBP (diastolic BP), -LR (negative likelihood ratio), +LR (positive likelihood ratio), sBP (systolic BP) CI (confidence interval), PI (prediction interval)

*Slade. The diagnostic accuracy of American College of Cardiology-American Heart Association blood pressure categories for identification of women and babies at risk. Am J Obstet Gynecol 2023*.

**Table S26:** Sensitivity and Specificity of ACC/AHA BP categories for placental abruption

		COMPARATOR	
BP measured at <20 weeks	Ref	Sensitivity	Specificity
**‘ELEVATED BP’ (as a new cut-off)**			
**Pooled**	**Normal (<120/80 mmHg)**	**0.25 [0.17, 0.34]**	**0.77 [0.69, 0.83]**
Greenberg		0.30 [0.24, 0.37]	0.72 [0.71, 0.72]
Qureshi		0.16 [0.10, 0.26]	0.84 [0.80, 0.87]
Sevene		0.25 [0.03, 0.80]	0.62 [0.22, 0.91]
Sutton 2020		0.27 [0.22, 0.33]	0.75 [0.74, 0.76]
**‘STAGE 1 HYPERTENSION’ (as a new cut-off)**			
**Pooled**	**Normal + Elevated BP (<130/80mmHg)**	**0.24 [0.15, 0.36]**	**0.79 [0.68, 0.86]**
Duffy		0.25 [0.24, 0.26]	0.84 [0.84, 0.84]
Greenberg		0.18 [0.13, 0.24]	0.86 [0.85, 0.86]
Huai		0.35 [0.23, 0.51]	0.66 [0.60, 0.70]
McLaren		0.50 [0.13, 0.87]	0.50 [0.44, 0.56]
Qureshi		0.12 [0.07, 0.20]	0.91 [0.87, 0.93]
Sabol		0.26 [0.24, 0.28]	0.77 [0.76, 0.77]
Sevene		0.25 [0.03, 0.80]	0.62 [0.22, 0.91]
Sutton 2020		0.10 [0.07, 0.14]	0.89 [0.89, 0.89]
**‘STAGE 2 HYPERTENSION’ (existing cut-off)**			
**Pooled**	**Normal + Elevated BP +Stage 1 HTN (<140/90mmHg)**	**0.10 [0.05, 0.19]**	**0.93 [0.86, 0.96]**
Greenberg		0.11 [0.07, 0.16]	0.93 [0.93, 0.94]
McLaren		0.36 [0.22, 0.53]	0.93 [0.93, 0.94]
Qureshi		0.30 [0.01, 0.09]	0.99 [0.97, 1.00]
Sabol		0.12 [0.06, 0.25]	0.90 [0.88, 0.91]
Sevene		0.25 [0.03, 0.80]	0.88 [0.40, 0.99]
Sutton 2020		0.06 [0.04, 0.10]	0.94 [0.94, 0.95]

*Slade. The diagnostic accuracy of American College of Cardiology-American Heart Association blood pressure categories for identification of women and babies at risk. Am J Obstet Gynecol 2023*.

**Table S27:** Pooled relative risk and mean likelihood ratios for Cesarean, according to BP cut-offs at <20 weeks, determined by ACC/AHA BP categories

		COMPARATOR		
BP measured at <20 weeks	Ref	RR [95% CI] I^2^, (95% PI)	-LR	+LR
**Diagnostic threshold: sBP 120mmHg (with dBP <80mmHg)**				
**Pooled**	**Normal (<120/80 mmHg)**	**1.3 [1.2, 1.4] I^2^=0%, (1.0, 1.6)**	**0.96 [0.93, 0.98]**	**1.28 [1.13, 1.45]**
Bellad		1.3 [1.2, 1.5]	0.98 [0.97, 0.99]	1.44 [1.21, 1.71]
Qureshi		1.2 [1.1, 1.4]	0.96 [0.94, 0.99]	1.23 [1.08, 1.40]
Rosner		1.3 [0.9, 1.8]	0.88 [0.75, 1.04]	1.29 [0.95, 1.76]
Sevene		0.9 [0.3, 2.6]	1.01 [0.90, 1.13]	0.94 [0.37, 2.43]
**Diagnostic threshold: sBP 130mmHg or dBP 80mmHg**				
**Pooled**	**Normal + ElevatedBP (<130/80 mmHg)**	**1.3 [1.1, 1.6] I^2^=99%, (1.0, 1.9)**	**0.94 [0.90, 0.97]**	**1.46 [1.26, 1.69]**
Bellad		1.5 [1.3, 1.8]	0.98 [0.97, 0.99]	1.66 [1.32, 2.08]
Bello		1.5 [1.5, 1.5]	0.90 [0.89, 0.90]	1.64 [1.60, 1.68]
Darwin		1.4 [1.3, 1.6]	0.89 [0.85, 0.93]	1.52 [1.32, 1.75]
Duffy		1.1 [1.1, 1.1]	0.97 [0.96, 0.97]	1.12 [1.10, 1.13]
Qureshi		1.2 [1.1, 1.4]	0.98 [0.96, 0.99]	1.26 [1.06, 1.49]
Sabol		1.4 [1.3, 1.6]	0.88 [0.84, 0.92]	1.57 [1.37, 1.80]
Sevene		0.2 [0.0, 3.5]	1.05 [1.01, 1.09]	0.22 [0.01, 3.43]
**Diagnostic threshold: sBP 140mmHg or dBP 90mmHg**				
**Pooled**	**Normal + Elevated BP +Stage 1 HTN (<140/90 mmHg)**	**1.7 [1.6, 1.8] I^2^=27%, (1.5, 1.9)**	**0.96 [0.93, 0.98]**	**2.31 [2.06, 2.55]**
Bellad		2.1 [1.6, 2.8]	0.99 [0.99, 1.00]	2.89 [1.76, 4.74]
Bello		1.7 [1.7, 1.8]	0.95 [0.95, 0.96]	2.30 [2.18, 2.41]
Darwin		1.6 [1.4, 1.8]	0.91 [0.88, 0.94]	1.84 [1.51, 2.24]
Qureshi		1.5 [1.1, 2.1]	0.99 [0.99, 1.00]	1.77 [1.11, 2.81]
Rosner		1.1 [0.6, 2.1]	0.99 [0.93, 1.05]	1.22 [0.52, 2.86]
Sabol		1.6 [1.4, 1.8]	0.91 [0.89, 0.94]	2.07 [1.68, 2.55]
Sevene		0.8 [0.0, 11.9]	1.00 [0.97, 1.04]	0.74 [0.04, 12.12]

ACC (American College of Cardiology), AHA (American Heart Association), BP (blood pressure), dBP (diastolic BP), -LR (negative likelihood ratio), +LR (positive likelihood ratio), sBP (systolic BP), CI (confidence interval), PI (prediction interval)

*Slade. The diagnostic accuracy of American College of Cardiology-American Heart Association blood pressure categories for identification of women and babies at risk. Am J Obstet Gynecol 2023*.

**Table S28:** Sensitivity and Specificity of ACC/AHA BP categories for caesarean

		COMPARATOR	
BP measured at <20 weeks	Ref	Sensitivity	Specificity
**‘ELEVATED BP’ (as a new cut-off)**			
**Pooled**	**Normal (<120/80mmHg)**	**0.16 [0.08, 0.30]**	**0.87 [0.75, 0.94]**
Bellad		0.07 [0.06, 0.08]	0.95 [0.95, 0.95]
Qureshi		0.18 [0.16, 0.20]	0.86 [0.85, 0.87]
Rosner		0.37 [0.28, 0.46]	0.72 [0.66, 0.77]
Sevene		0.11 [0.04, 0.25]	0.89 [0.86, 0.91]
**‘STAGE 1 HYPERTENSION’ (as a new cut-off)**			
**Pooled**	**Normal + ElevatedBP (<130/80mmHg)**	**0.16 [0.09, 0.26]**	**0.89 [0.82, 0.94]**
Bellad		0.04 [0.04, 0.05]	0.97 [0.97, 0.98]
Bello		0.23 [0.22, 0.23]	0.86 [0.86, 0.86]
Darwin		0.26 [0.24, 0.29]	0.83 [0.81, 0.84]
Duffy		0.25 [0.25, 0.26]	0.77 [0.77, 0.78]
Qureshi		0.11 [0.09, 0.12]	0.92 [0.91, 0.92]
Sabol		0.27 [0.25, 0.30]	0.83 [0.81, 0.84]
Sevene		0.01 [0.00, 0.11]	0.94 [0.92, 0.95]
**‘STAGE 2 HYPERTENSION’ (existing cut-off)**			
**Pooled**	**Normal + Elevated BP +Stage 1 HTN (<140/90mmHg)**	**0.06 [0.03, 0.13]**	**0.97 [0.94, 0.99]**
Bellad		0.01 [0.01, 0.02]	1.00 [0.99, 1.00]
Bello		0.08 [0.08, 0.09]	0.96 [0.96, 0.97]
Darwin		0.17 [0.15, 0.20]	0.91 [0.89, 0.92]
Qureshi		0.08 [0.01, 0.48]	0.99 [0.99, 0.99]
Rosner		0.07 [0.03, 0.13]	0.95 [0.91, 0.97]
Sabol		0.16 [0.14, 0.18]	0.92 [0.91, 0.93]
Sevene		0.01 [0.00, 0.11]	0.98 [0.97, 0.99]

*Slade. The diagnostic accuracy of American College of Cardiology-American Heart Association blood pressure categories for identification of women and babies at risk. Am J Obstet Gynecol 2023*.

**Figure S1:**
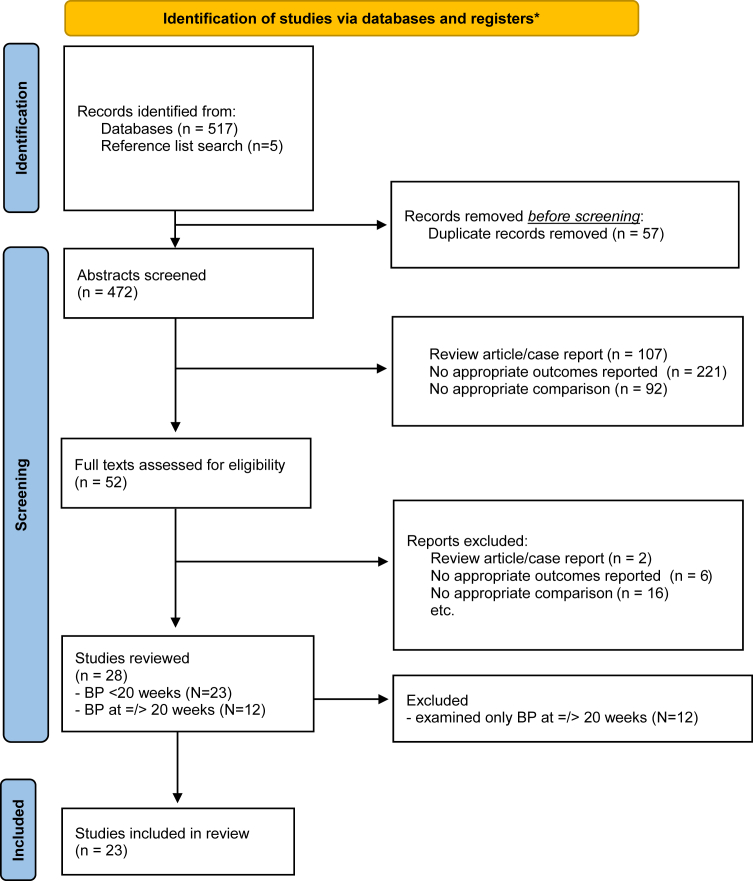
PRISMA flow diagram for study inclusion and exclusion *From:* Page MJ, McKenzie JE, Bossuyt PM, Boutron I, Hoffmann TC, Mulrow CD, et al. The PRISMA 2020 statement: an updated guideline for reporting systematic reviews. BMJ 2021;372:n71. doi: 10.1136/bmj.n71. For more information, visit: http://www.prisma-statement.org/ *Slade. The diagnostic accuracy of American College of Cardiology-American Heart Association blood pressure categories for identification of women and babies at risk. Am J Obstet Gynecol 2023*.

## Supplementary Data

AHA BP categories - 10 min

## References

[1] Magee L.A., Nathan H.L., Shennan A.H. (2021). Pregnancy outcomes and blood pressure visit-to-visit variability in three less-developed countries. BJOG.

[2] Davey D.A., MacGillivray I. (1988). The classification and definition of the hypertensive disorders of pregnancy. Am J Obstet Gynecol.

[3] Stone P., Cook D., Hutton J., Purdie G., Murray H., Harcourt L. (1995). Measurements of blood pressure, oedema and proteinuria in a pregnant population of New Zealand. Aust N Z J Obstet Gynaecol.

[4] von Dadelszen P., Payne B., Li J. (2011). Prediction of adverse maternal outcomes in pre-eclampsia: development and validation of the fullPIERS model. Lancet.

[5] Magee L.A., Brown M.A., Hall D.R. (2022). The 2021 International Society for the Study of Hypertension in Pregnancy classification, diagnosis & management recommendations for international practice. Pregnancy Hypertens.

[6] Whelton P.K., Carey R.M., Aronow W.S. (2018). 2017 ACC/AHA/AAPA/ABC/ACPM/AGS/APhA/ASH/ASPC/NMA/PCNA guideline for the prevention, detection, evaluation, and management of high blood pressure in adults: a report of the American College of Cardiology/American Heart Association Task Force on Clinical Practice guidelines. Hypertension.

[7] Scott G., Gillon T.E., Pels A., von Dadelszen P., Magee L.A. (2022). Guidelines-similarities and dissimilarities: a systematic review of international clinical practice guidelines for pregnancy hypertension. Am J Obstet Gynecol.

[8] Darwin K.C., Federspiel J.J., Schuh B.L., Baschat A.A., Vaught A.J. (2021). ACC-AHA Diagnostic Criteria for Hypertension in Pregnancy identifies patients at intermediate risk of adverse outcomes. Am J Perinatol.

[9] Greenberg V.R., Silasi M., Lundsberg L.S. (2021). Perinatal outcomes in women with elevated blood pressure and stage 1 hypertension. Am J Obstet Gynecol.

[10] Hauspurg A., Sutton E.F., Catov J.M., Caritis S.N. (2018). Aspirin effect on adverse pregnancy outcomes associated with stage 1 hypertension in a high-risk cohort. Hypertension.

[11] Hu J., Li Y., Zhang B. (2019). Impact of the 2017 ACC/AHA guideline for high blood pressure on evaluating gestational hypertension-associated risks for newborns and mothers. Circ Res.

[12] Liu J., Tao L., Cao Y. (2020). Stage 1 hypertension defined by the 2017 American College of Cardiology/American Heart Association guideline and risk of adverse birth outcomes in Eastern China. J Hypertens.

[13] Reddy M., Rolnik D.L., Harris K., Challenging the definition of hypertension in pregnancy: a retrospective cohort study (2020). Am J Obstet Gynecol.

[14] Sabol B.A., Porcelli B., Diveley E. (2021). Defining the risk profile of women with stage 1 hypertension: a time to event analysis. Am J Obstet Gynecol MFM.

[15] Brown M.A., Magee L.A., Kenny L.C. (2018). The hypertensive disorders of pregnancy: ISSHP classification, diagnosis & management recommendations for international practice. Pregnancy Hypertens.

[16] University of Bristol. Quadas-2 [Internet]. 2 | Bristol Medical School: Population Health Sciences | University of Bristol. University of Bristol; 2014. Available at: https://wwwbristolacuk/population-health-sciences/projects/quadas/quadas-2/ Accessed Feb 21 2021.

[17] Whiting P.F., Rutjes A.W., Westwood M.E. (2011). QUADAS-2: a revised tool for the quality assessment of diagnostic accuracy studies. Ann Intern Med.

[18] Greenland S., Robins J.M. (1985). Estimation of a common effect parameter from sparse follow-up data. Biometrics.

[19] Higgins J.P., Altman D.G., Gøtzsche P.C. (2011). The Cochrane Collaboration’s tool for assessing risk of bias in randomised trials. BMJ.

[20] Zwinderman A.H., Bossuyt P.M. (2008). We should not pool diagnostic likelihood ratios in systematic reviews. Stat Med.

[21] Reitsma J.B., Glas A.S., Rutjes A.W., Scholten R.J., Bossuyt P.M., Zwinderman A.H. (2005). Bivariate analysis of sensitivity and specificity produces informative summary measures in diagnostic reviews. J Clin Epidemiol.

[22] Harbord R.M., Deeks J.J., Egger M., Whiting P., Sterne J.A. (2007). A unification of models for meta-analysis of diagnostic accuracy studies. Biostatistics.

[23] Jaeschke R., Guyatt G.H., Sackett D.L. (1994). Users’ guides to the medical literature. III. How to use an article about a diagnostic test. B. What are the results and will they help me in caring for my patients? Jama, Sierra Leone: The Evidence-Based Medicine Working Group.

[24] Duval S., Tweedie R.R. (2000). Trim and fill: a simple funnel-plot-based method of testing and adjusting for publication bias in meta-analysis. Biometrics.

[25] Balduzzi S., Rücker G., Schwarzer G. (2019). How to perform a meta-analysis with R: a practical tutorial. Evid Based Ment Health.

[26] Doebler P, Holling H. Meta-analysis of diagnostic accuracy with mada. 2020. Available at: https://cran.r-project.org/web/packages/mada/vignettes/mada.pdf. Accessed May 12, 2021.

[27] mada DP. Meta-analysis of diagnostic accuracy. R package version 0.5.10. 2020. Available at: https://CRANR-projectorg/package=mada. Accessed May 12, 2021.

[28] Rosner J.Y., Gutierrez M., Dziadosz M. (2017). Prehypertension in early pregnancy: what is the significance?. Am J Perinatol.

[29] Bellad M.B., Goudar S.S., Mallapur A.A. (2020). Community level interventions for pre-eclampsia (CLIP) in India: a cluster randomised controlled trial. Pregnancy Hypertens.

[30] Bello N.A., Zhou H., Cheetham T.C. (2021). Prevalence of hypertension among pregnant women when using the 2017 American College of Cardiology/American Heart Association blood pressure guidelines and association with maternal and fetal outcomes. JAMA Netw Open.

[31] Duffy J.Y., Getahun D., Chen Q., Fong A. (2021). Pregnancy outcomes associated with a single elevated blood pressure before 20 weeks of gestation. Obstet Gynecol.

[32] González-Valencia D.P., Valero-Rubio S.Y., Fernando Grillo-Ardila C. (2020). Prehypertension as a risk factor for the development of perinatal complications: retrospective cohort study. Pregnancy Hypertens.

[33] Hauspurg A., Parry S., Mercer B.M. (2019). Blood pressure trajectory and category and risk of hypertensive disorders of pregnancy in nulliparous women. Am J Obstet Gynecol.

[34] Yang X., Chen D., He B., Cheng W. (2021). NRP1 and MMP9 are dual targets of RNA-binding protein QKI5 to alter VEGF-R/ NRP1 signalling in trophoblasts in preeclampsia. J Cell Mol Med.

[35] Huai J., Lin L., Juan J. (2021). Preventive effect of aspirin on preeclampsia in high-risk pregnant women with stage 1 hypertension. J Clin Hypertens (Greenwich).

[36] Li Q., Zheng L., Gu Y. (2022). Early pregnancy stage 1 hypertension and high mean arterial pressure increased risk of adverse pregnancy outcomes in Shanghai, China. J Hum Hypertens.

[37] McLaren R.A., Atallah F., Persad V.V.D. (2021). Pregnancy outcomes among women with American College of Cardiology- American Heart Association defined hypertension. J Matern Fetal Neonatal Med.

[38] Nagao T., Saito K., Yamanaka M. (2021). Prehypertension in early pregnancy is a risk factor for hypertensive disorders during pregnancy: a historical cohort study in Japan. Hypertens Pregnancy.

[39] Qureshi R.N., Sheikh S., Hoodbhoy Z. (2020). Community-level interventions for pre-eclampsia (CLIP) in Pakistan: a cluster randomised controlled trial. Pregnancy Hypertens.

[40] Rosner J.Y., Gutierrez M., Dziadosz M. (2019). Prehypertension in early versus late pregnancy. J Matern Fetal Neonatal Med.

[41] Sevene E., Sharma S., Munguambe K. (2020). Community-level interventions for pre-eclampsia (CLIP) in Mozambique: a cluster randomised controlled trial. Pregnancy Hypertens.

[42] Sutton E.F., Hauspurg A., Caritis S.N., Powers R.W., Catov J.M. (2018). Maternal outcomes associated with lower range stage 1 hypertension. Obstet Gynecol.

[43] Sutton E.F., Rogan S.C., Lopa S., Sharbaugh D., Muldoon M.F., Catov J.M. (2020). Early pregnancy blood pressure elevations and risk for maternal and neonatal morbidity. Obstet Gynecol.

[44] Wu D.D., Gao L., Huang O. (2020). Increased adverse pregnancy outcomes associated with stage 1 hypertension in a low-risk cohort: evidence from 47 874 cases. Hypertension.

[45] Yang S.W., Cho S.H., Kang Y.S. (2019). Usefulness of uterine artery Doppler velocimetry as a predictor for hypertensive disorders in pregnancy in women with prehypertension before 20 weeks gestation. PLoS One.

[46] He D., Wu S., Zhao H., Zheng Z., Zhang W. (2018). High normal blood pressure in early pregnancy also contribute to early onset preeclampsia and severe preeclampsia. Clin Exp Hypertens.

[47] Bello N.A., Zhou H., Cheetham T.C. (2021). Prevalence of hypertension among pregnant women when using the 2017 American College of Cardiology/American Heart Association blood pressure guidelines and association with maternal and fetal outcomes. Obstet Gynecol Surv.

[48] Bramham K., Parnell B., Nelson-Piercy C., Seed P.T., Poston L., Chappell L.C. (2014). Chronic hypertension and pregnancy outcomes: systematic review and meta-analysis. BMJ.

[49] Al Khalaf S.Y., O’Reilly É.J., Barrett P.M. (2021). Impact of chronic hypertension and antihypertensive treatment on adverse perinatal outcomes: systematic review and meta-analysis. J Am Heart Assoc.

[50] Gestational hypertension and preeclampsia (2020). ACOG Practice Bulletin, Number 222. Obstet Gynecol.

[51] Antwi E., Amoakoh-Coleman M., Vieira D.L. (2020). Systematic review of prediction models for gestational hypertension and preeclampsia. PLoS One.

[52] Wright D., Wright A., Nicolaides K.H. (2020). The competing risk approach for prediction of preeclampsia. Am J Obstet Gynecol.

[53] Magee L.A., von Dadelszen P., Singer J. (2016). The CHIPS randomized controlled trial (control of hypertension in pregnancy study): is severe hypertension just an elevated blood pressure?. Hypertension.

[54] Tita A.T., Szychowski J.M., Boggess K. (2022). Treatment for mild chronic hypertension during pregnancy. N Engl J Med.

[55] Brown M.A., Magee L.A., Kenny L.C. (2018). Hypertensive disorders of pregnancy: ISSHP classification, diagnosis, and management recommendations for international practice. Hypertension.

[56] American College of Obstetricians and Gynecologists' Committee on Practice Bulletins—Obstetrics (2019). ACOG Practice Bulletin No. 203: chronic hypertension in pregnancy. Obstet Gynecol.

[57] National Collaborating Centre for Women’s and Children’s Health (UK). Hypertension in Pregnancy: The Management of Hypertensive Disorders During Pregnancy. London: RCOG Press; 2010. 22220321

[58] Cluver C., Tong S. (2021). Revisiting blood pressure thresholds to define hypertension during pregnancy: is 140/90 mmHg too high?. Lancet Glob Health.

[59] Tan M.Y., Syngelaki A., Poon L.C. (2018). Screening for pre-eclampsia by maternal factors and biomarkers at 11-13 weeks’ gestation. Ultrasound Obstet Gynecol.

