# Supplementary figures and images for: American College of Cardiology and American Heart Association blood pressure categories—a systematic review of the relationship with adverse pregnancy outcomes in the first half of pregnancy
Source: Am J Obstet Gynecol. 2023 Apr;228(4):418–429.e34. doi: 10.1016/j.ajog.2022.10.004 (PMC10239058; doi:10.1016/j.ajog.2022.10.004)

## Slide 1
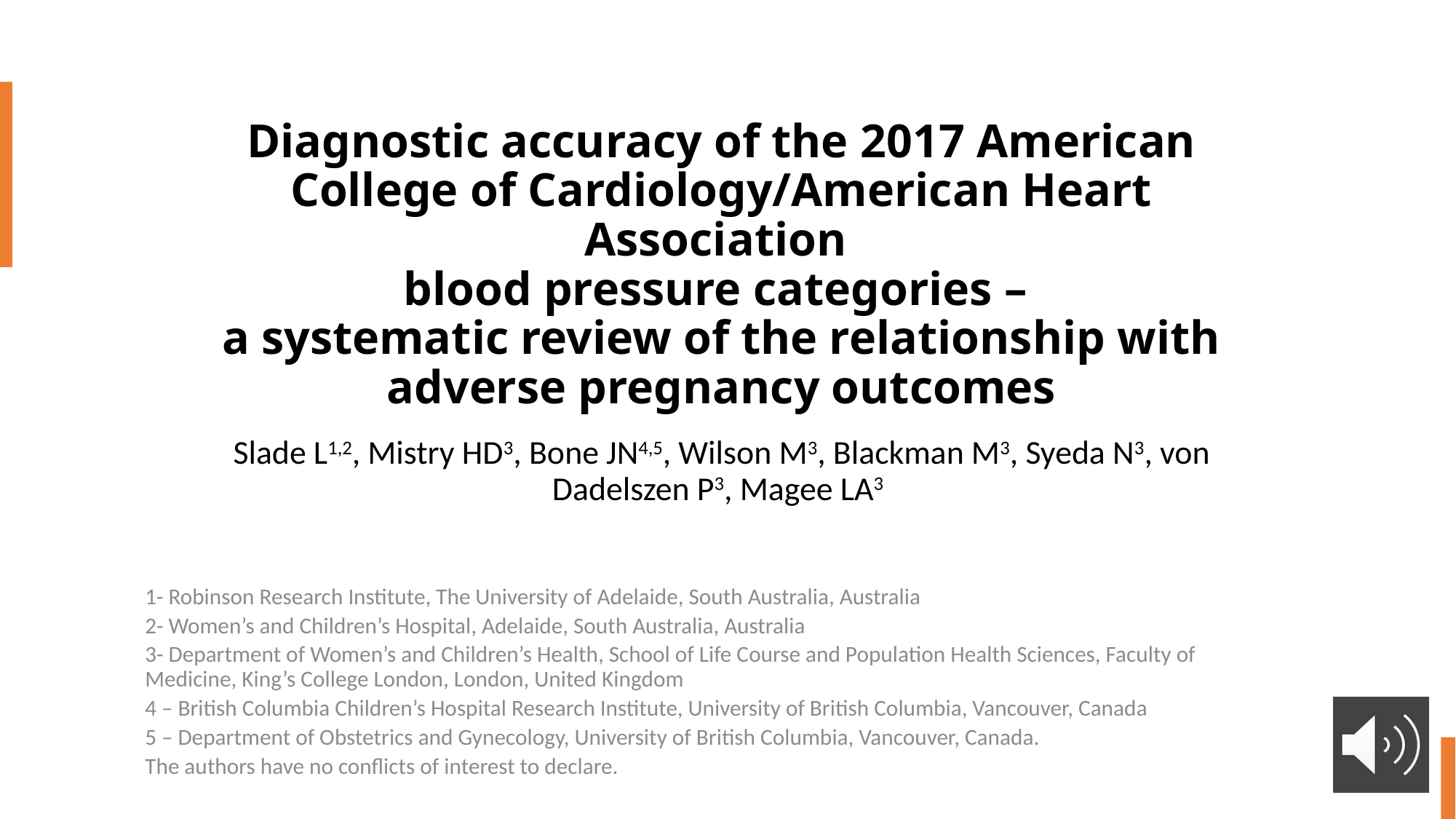

## Slide 2
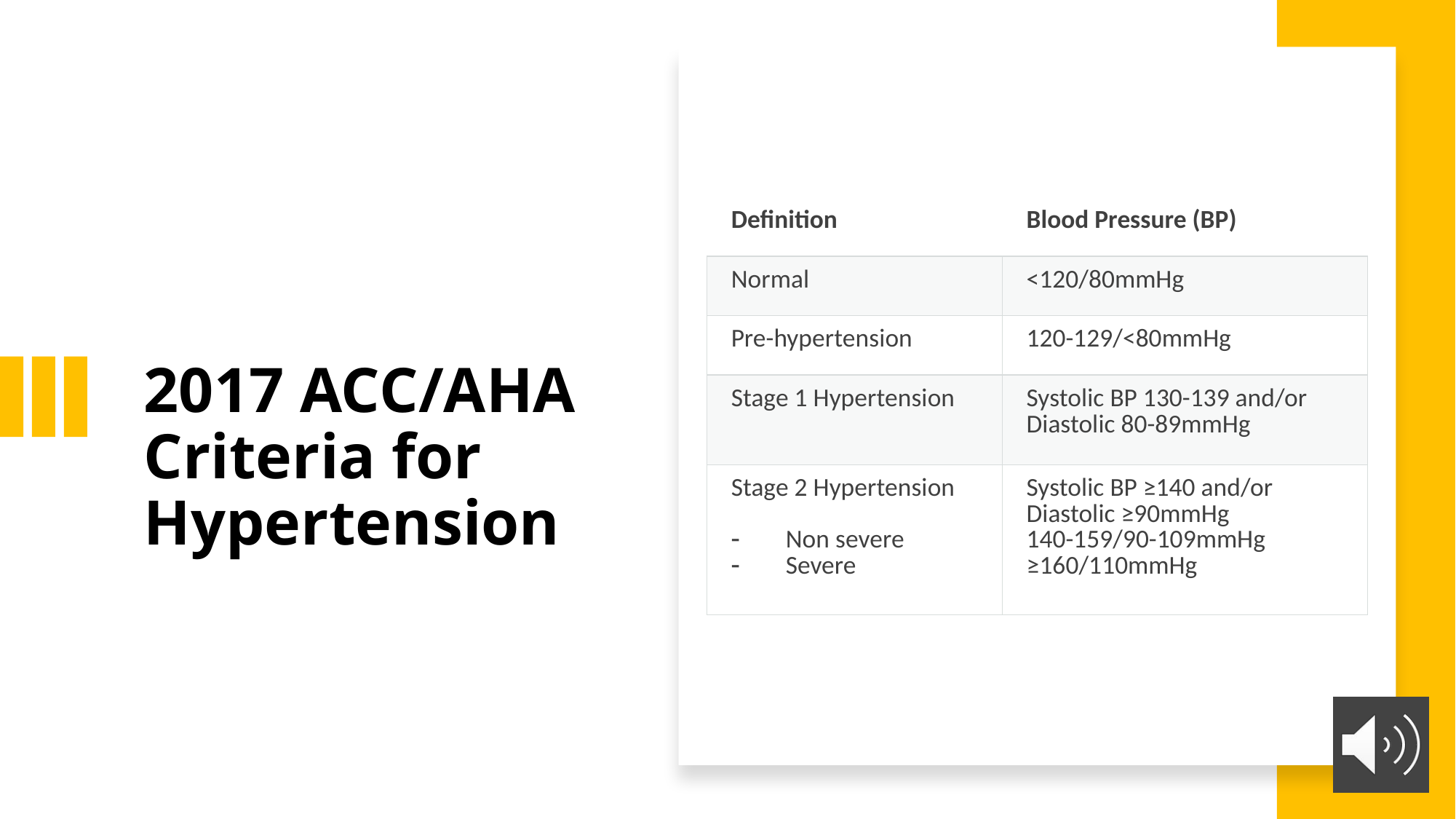

## Slide 3
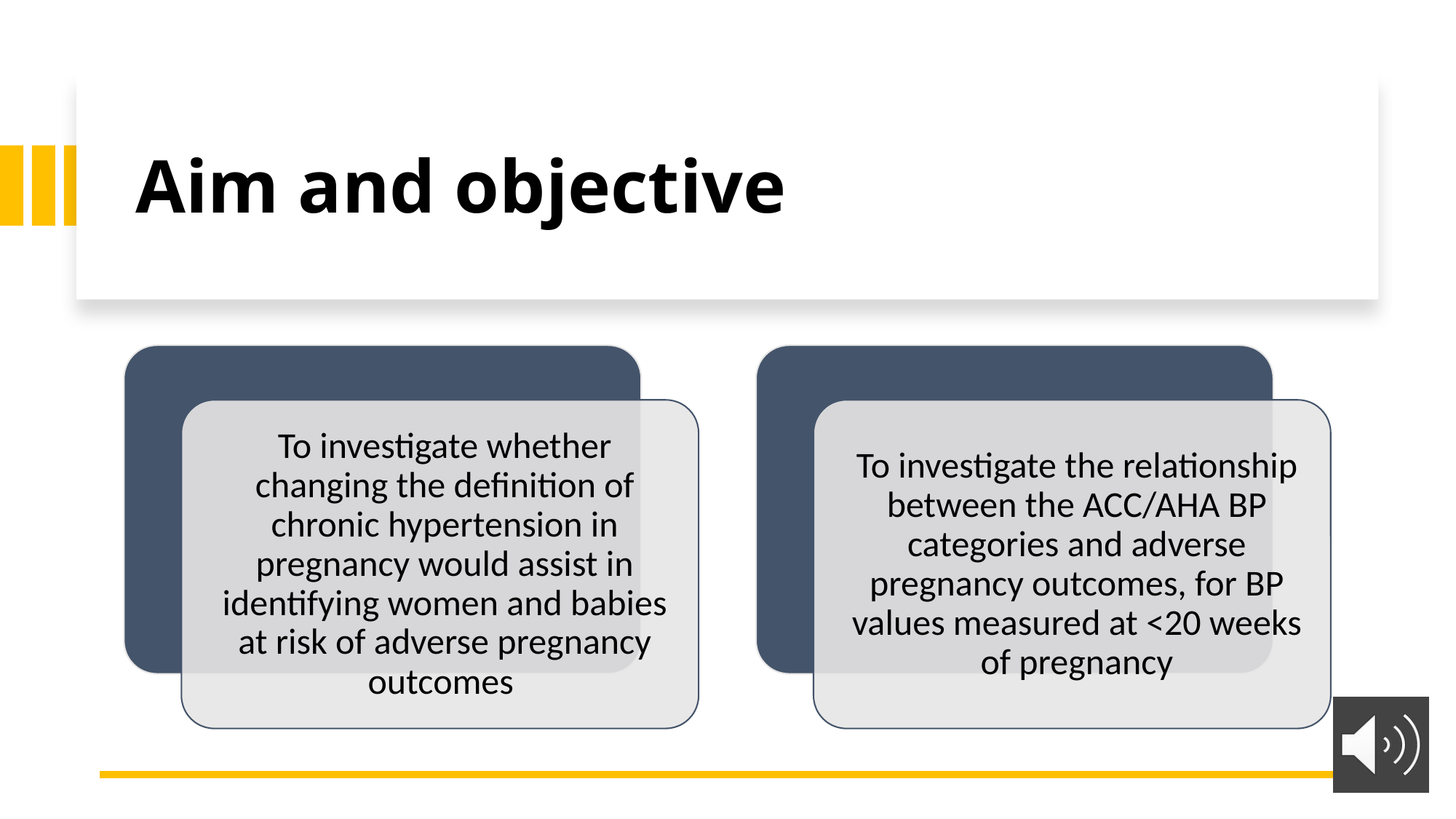

## Slide 4
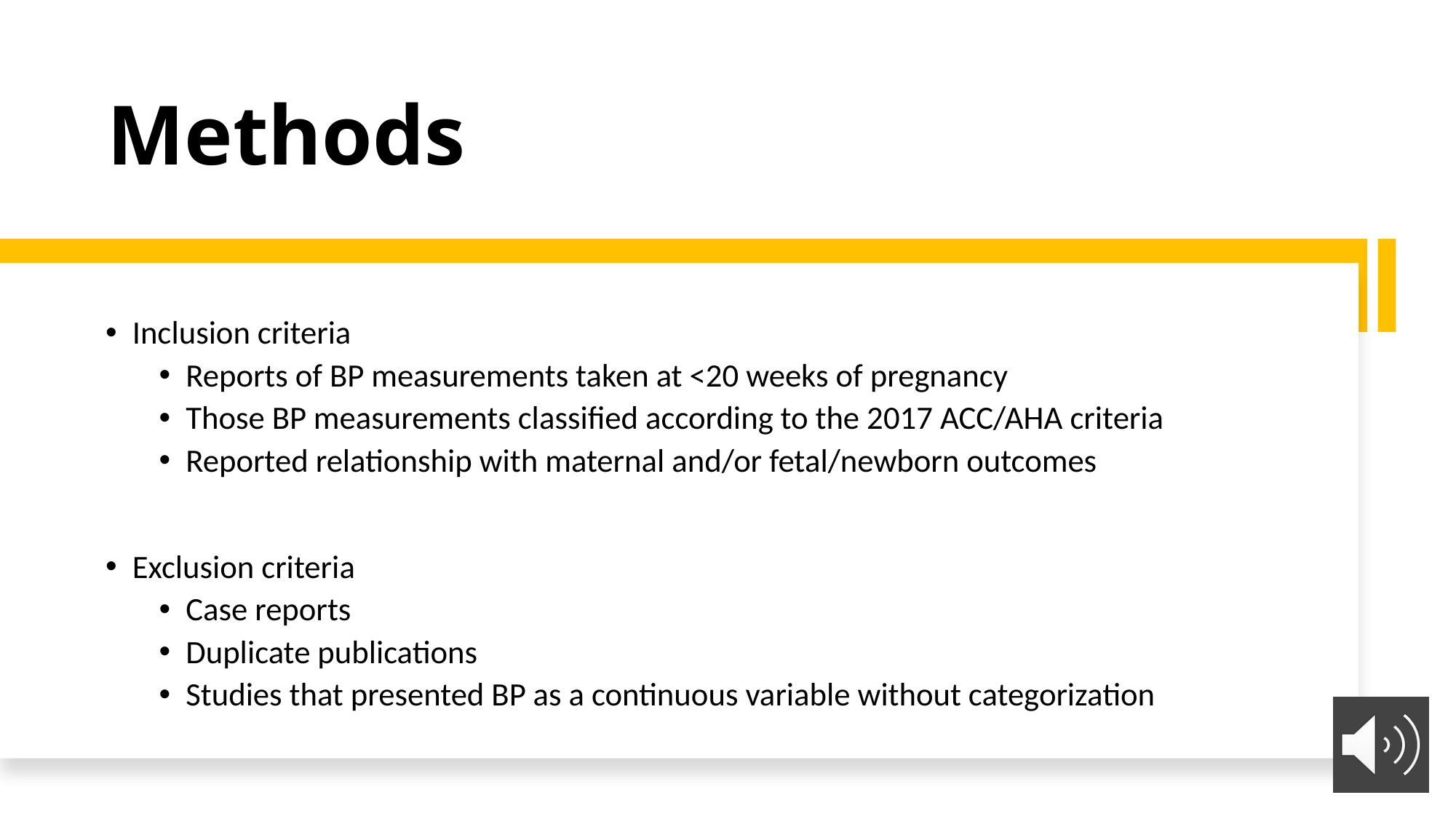

## Slide 5
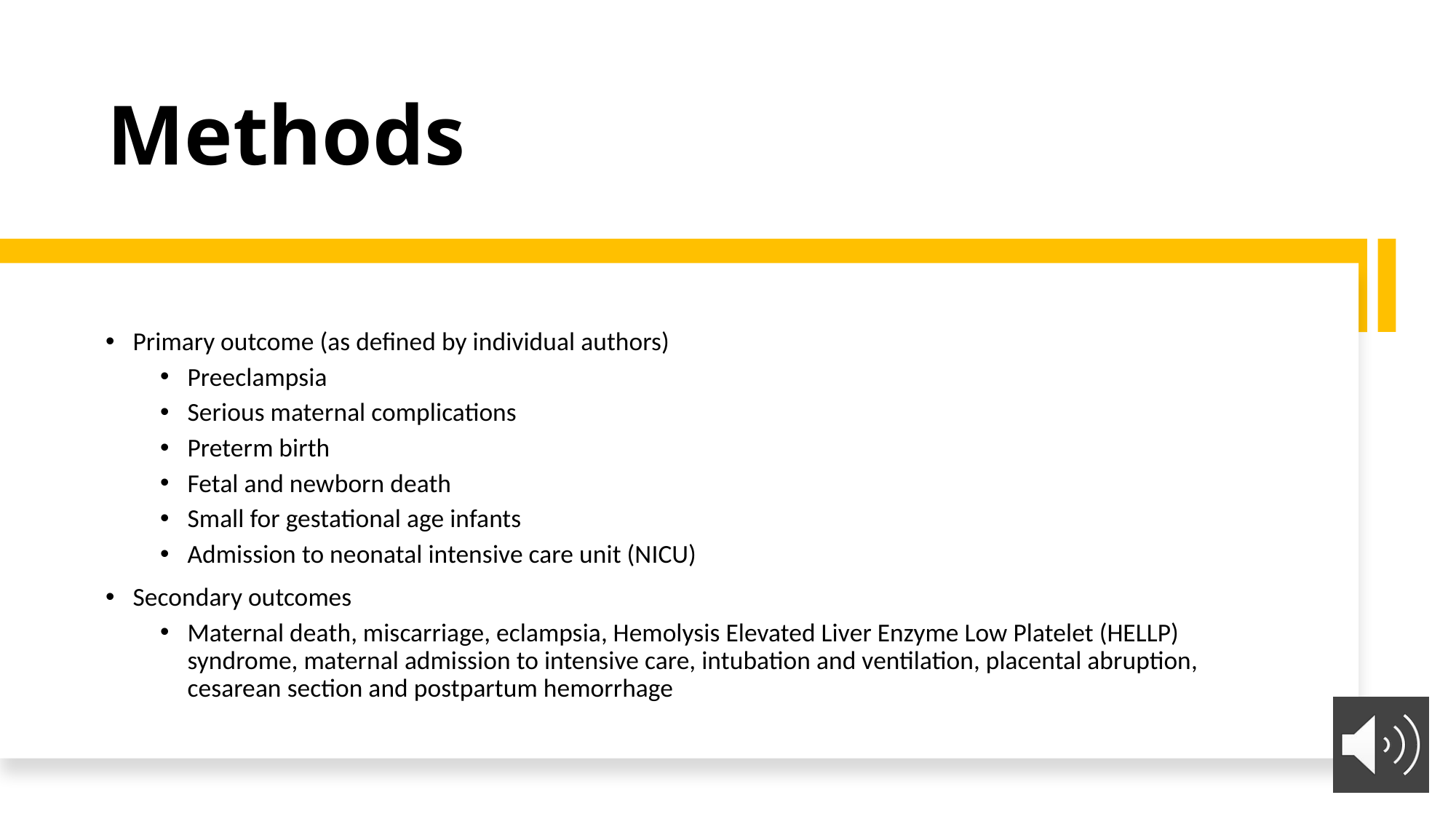

## Slide 6
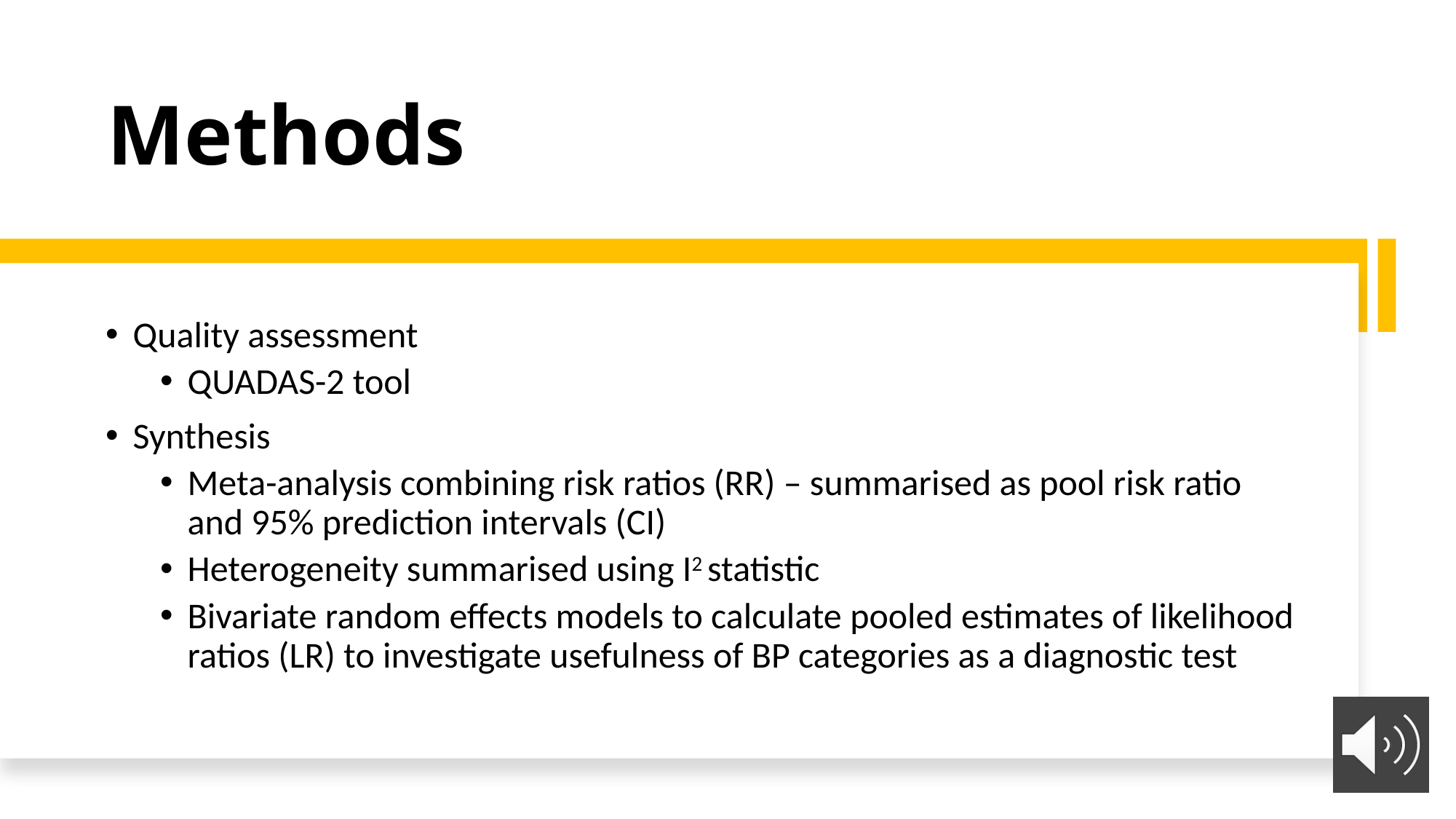

## Slide 7
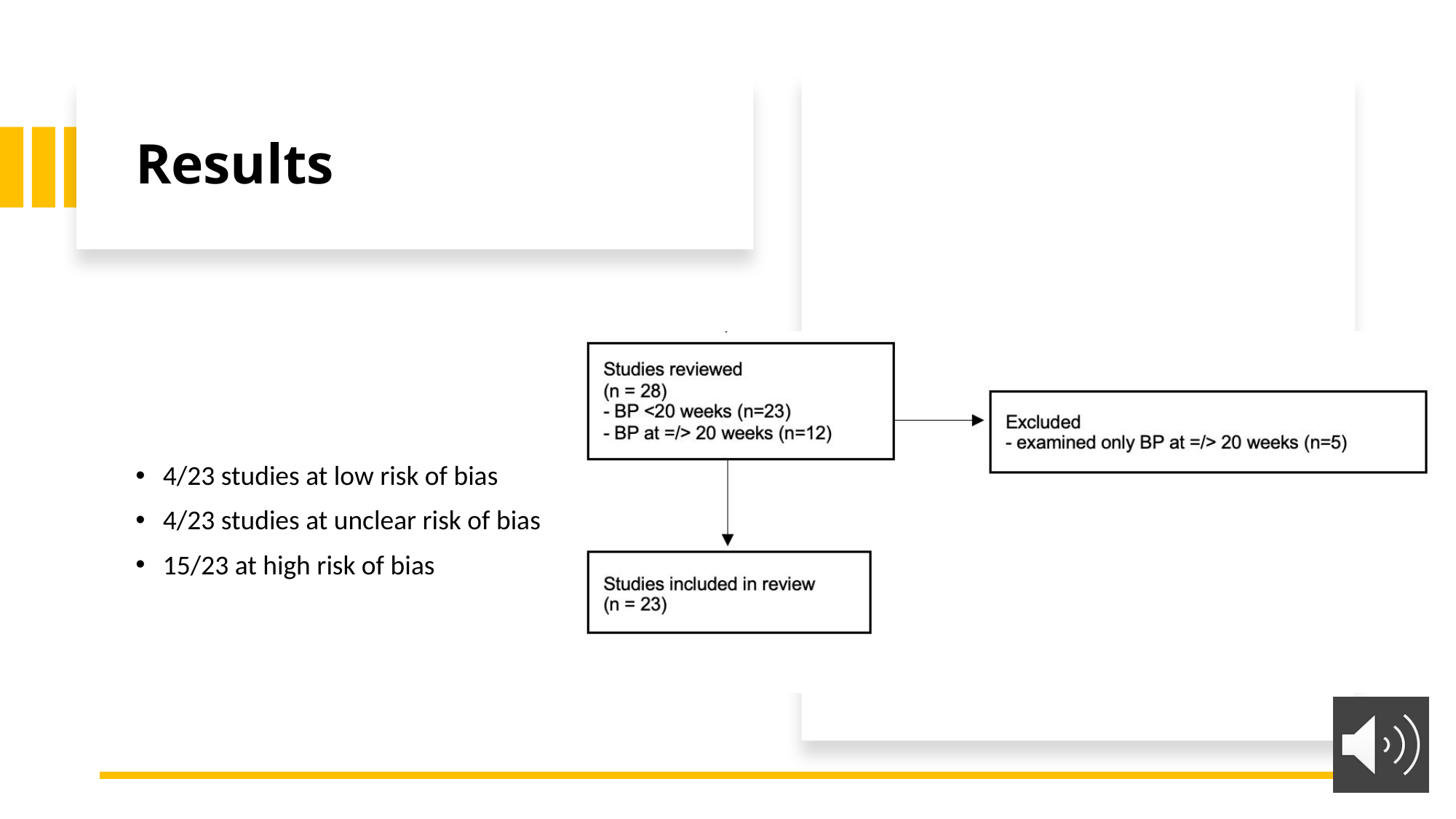

## Slide 8
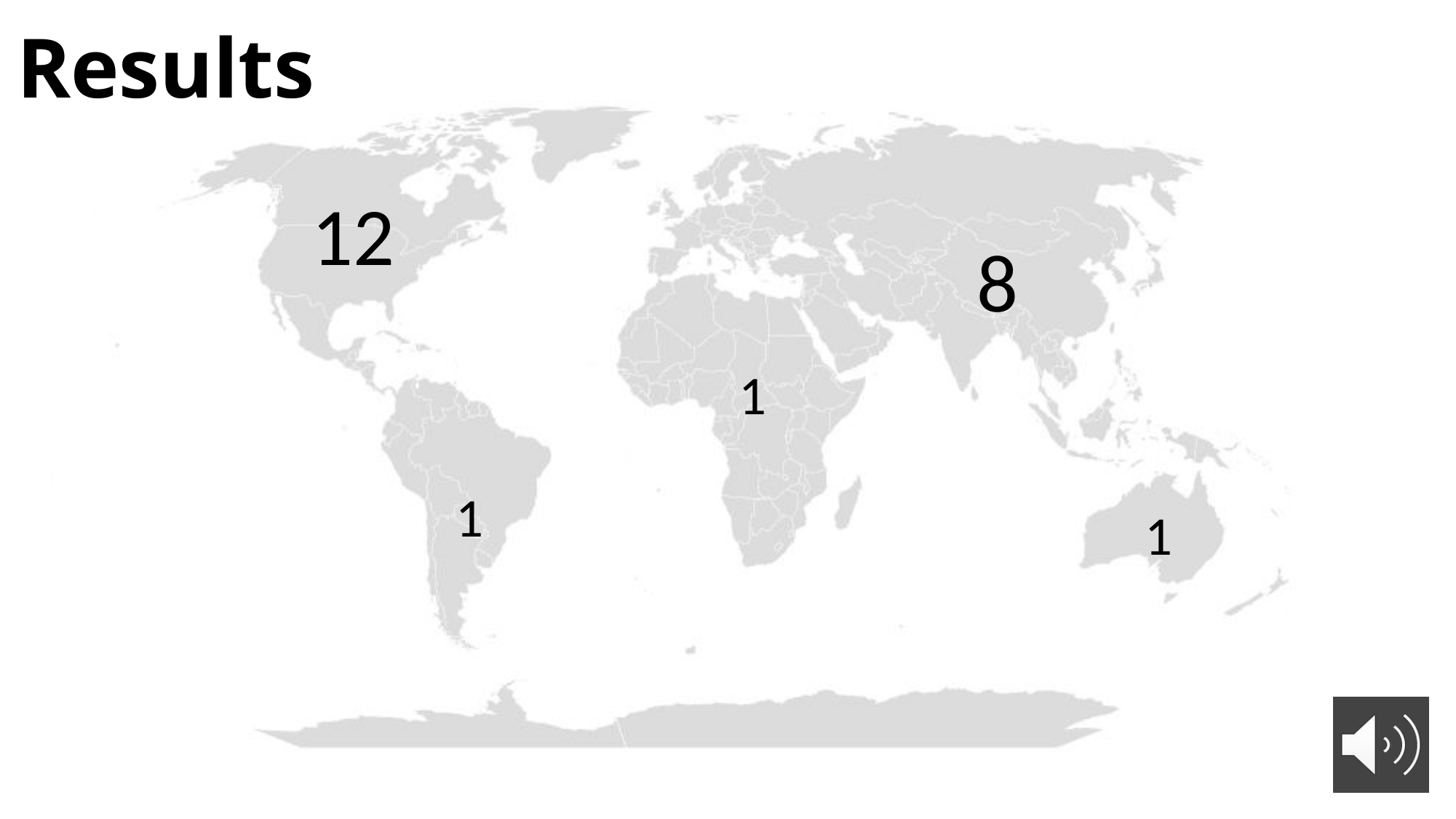

## Slide 9
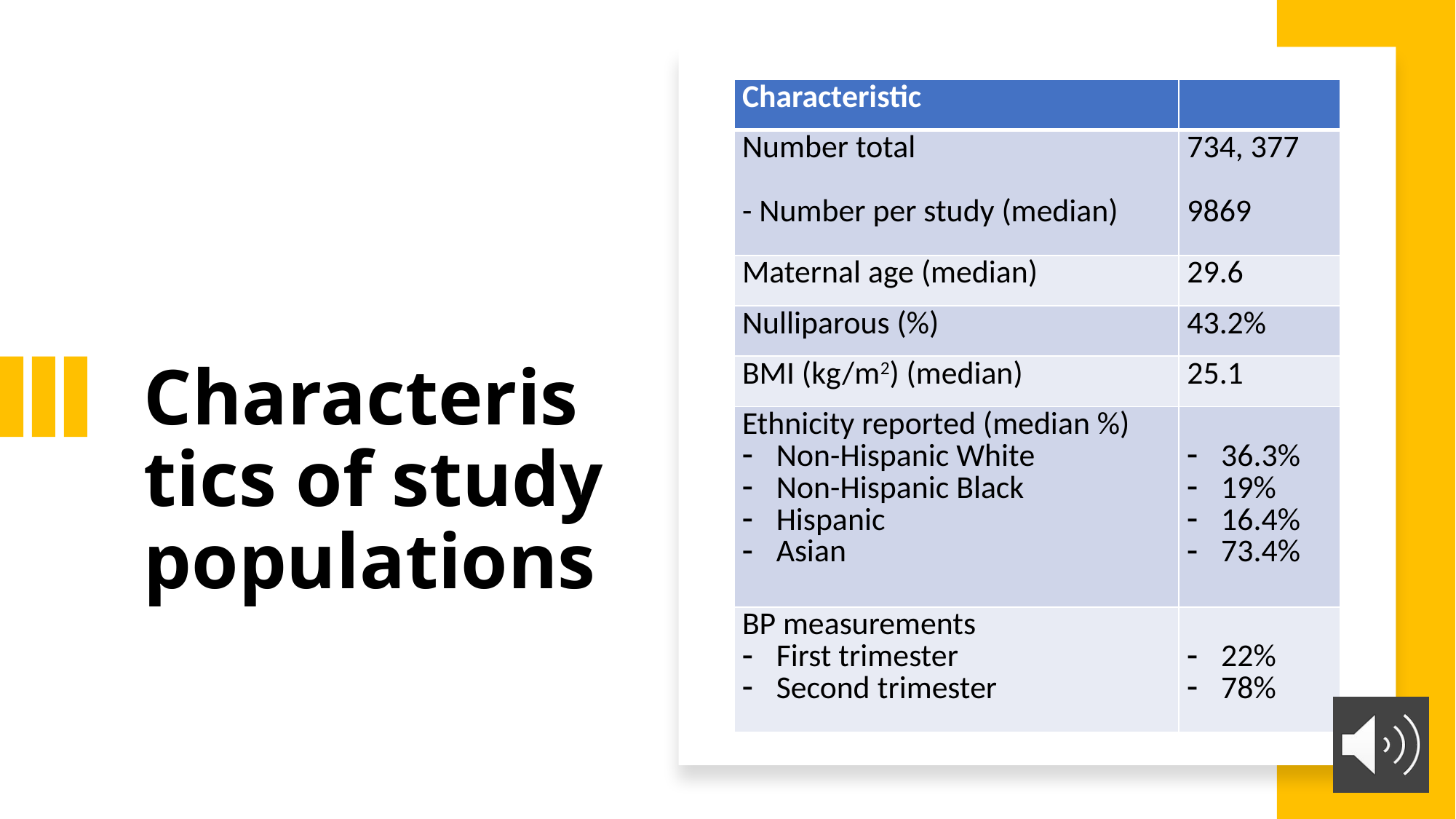

## Slide 10
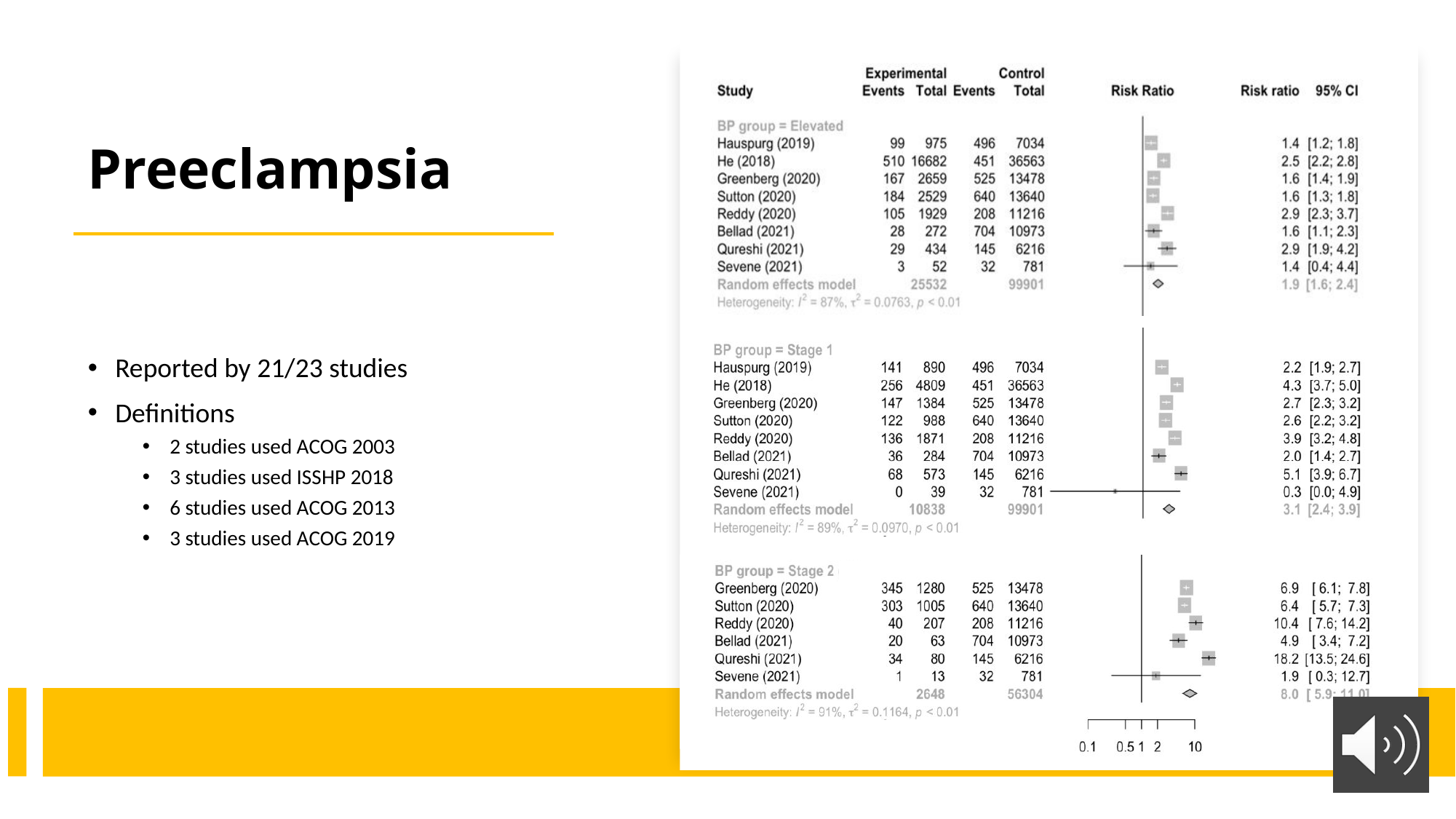

## Slide 11
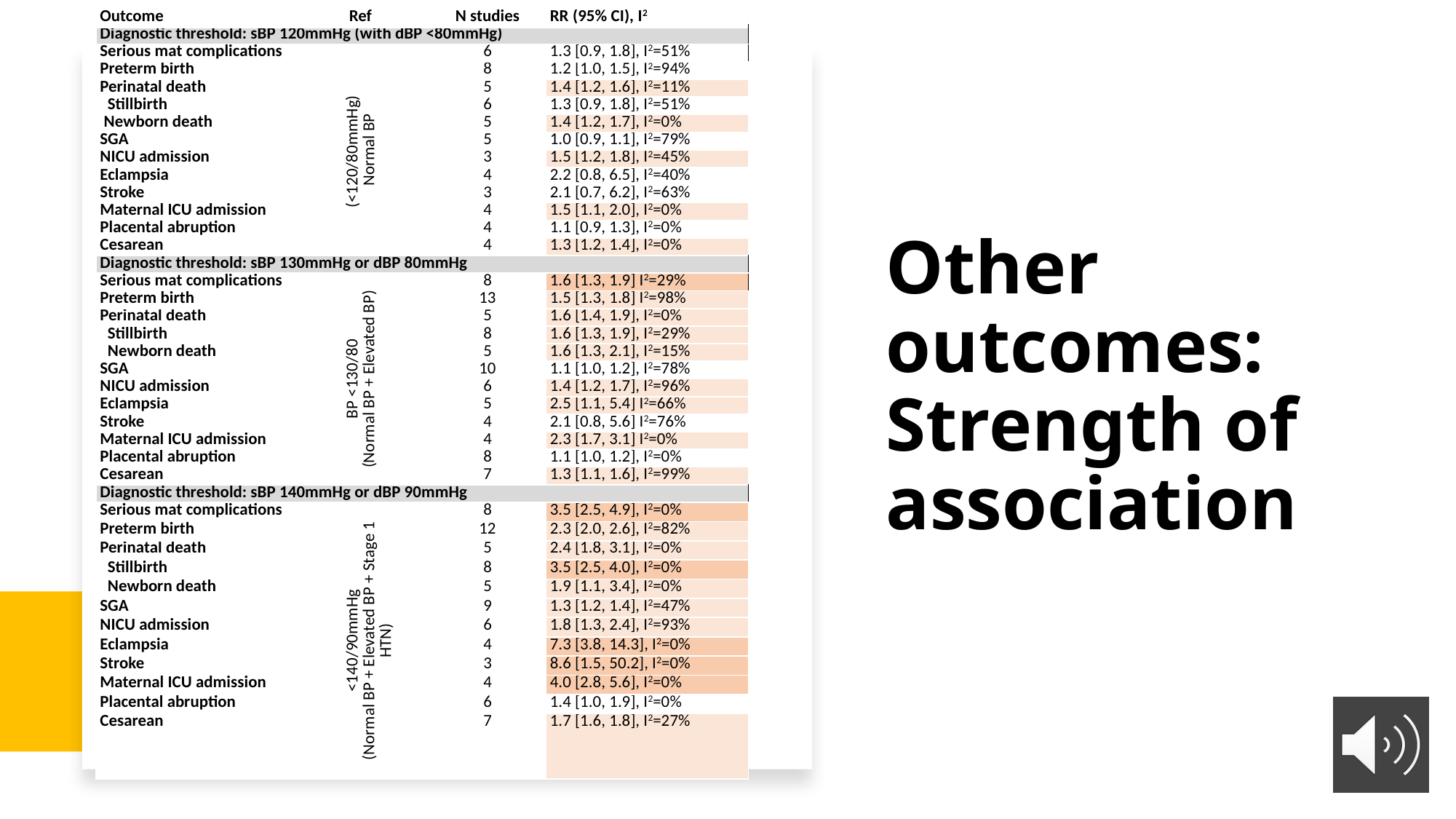

## Slide 12
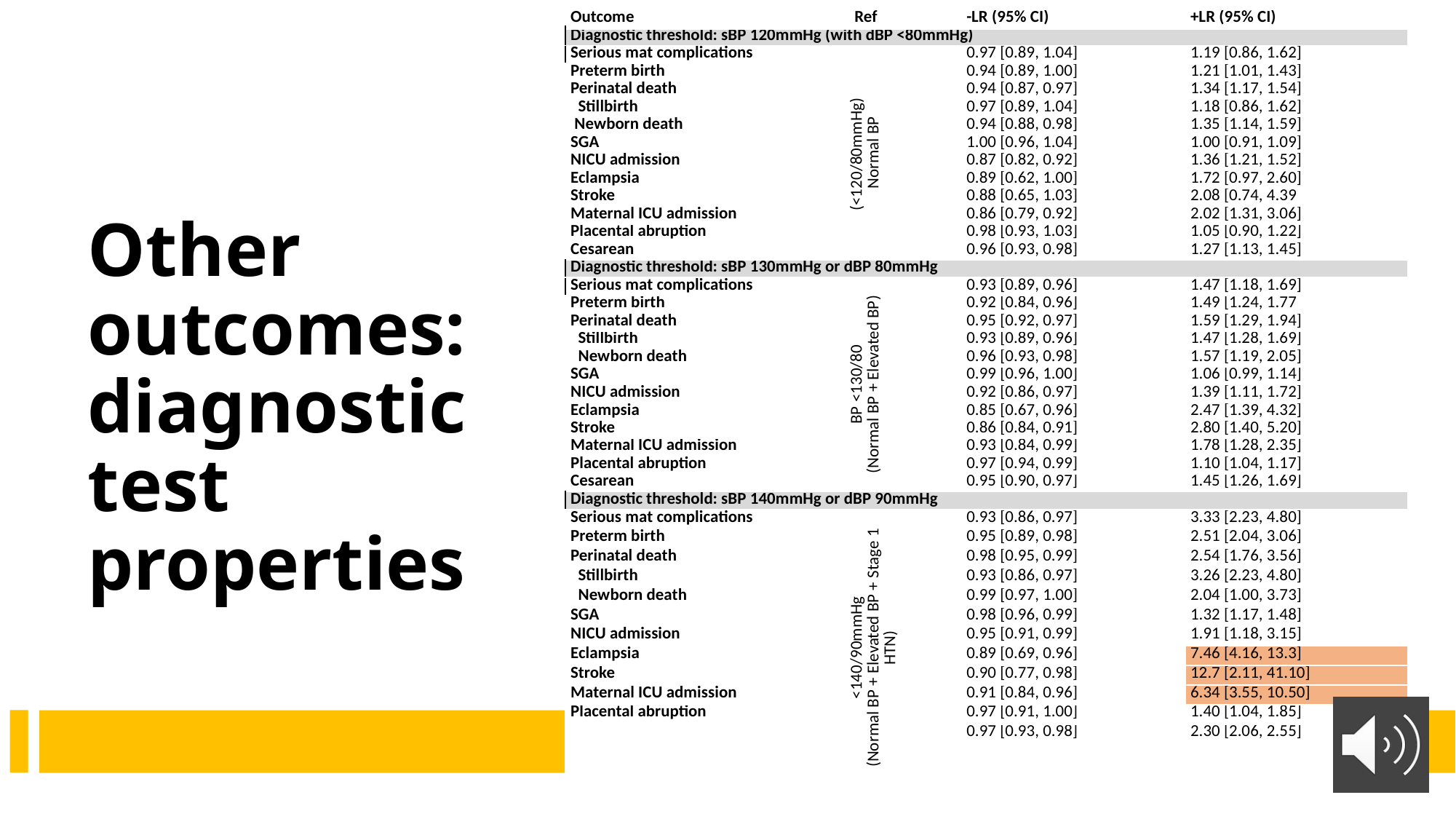

## Slide 13
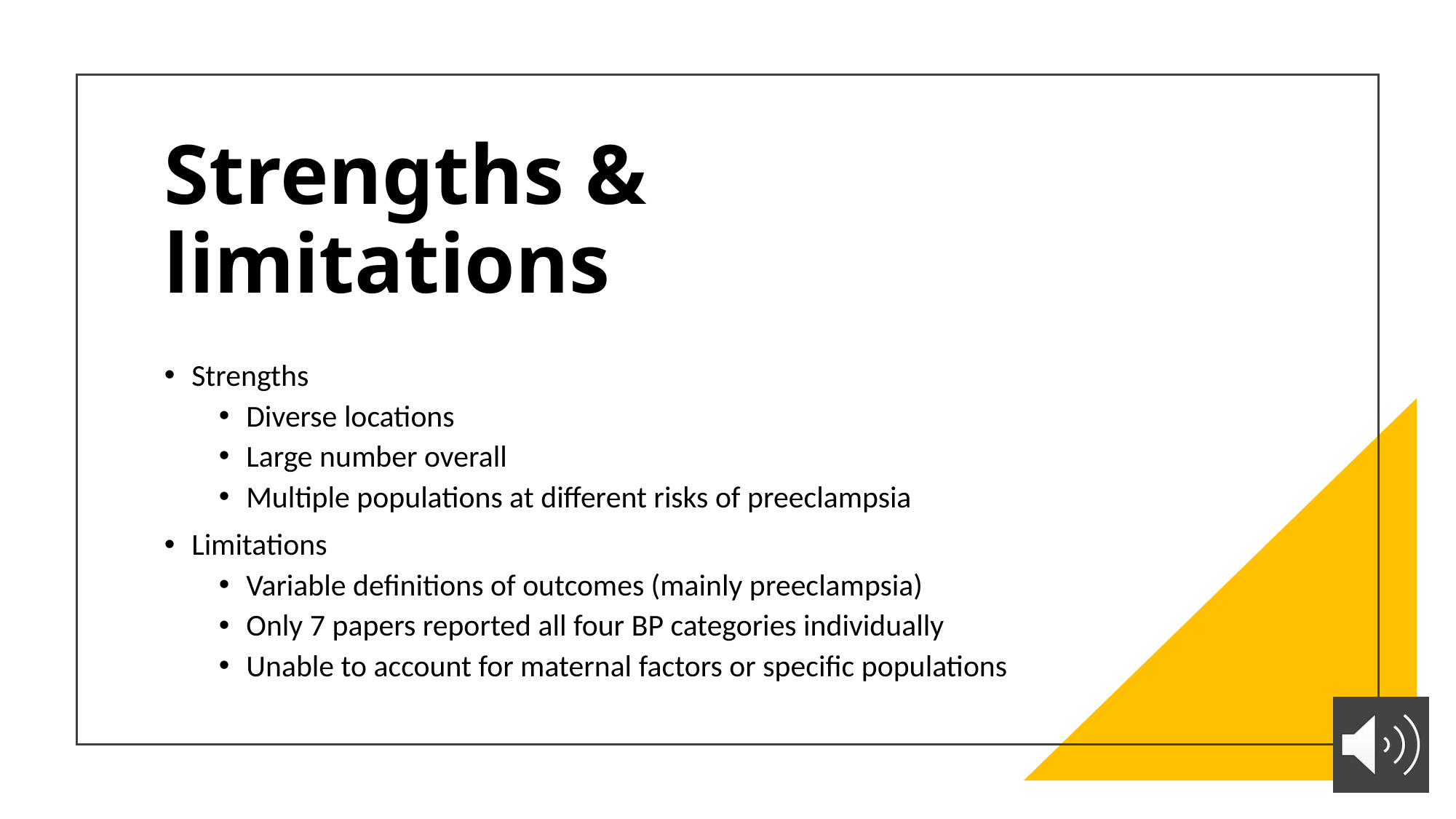

## Slide 14
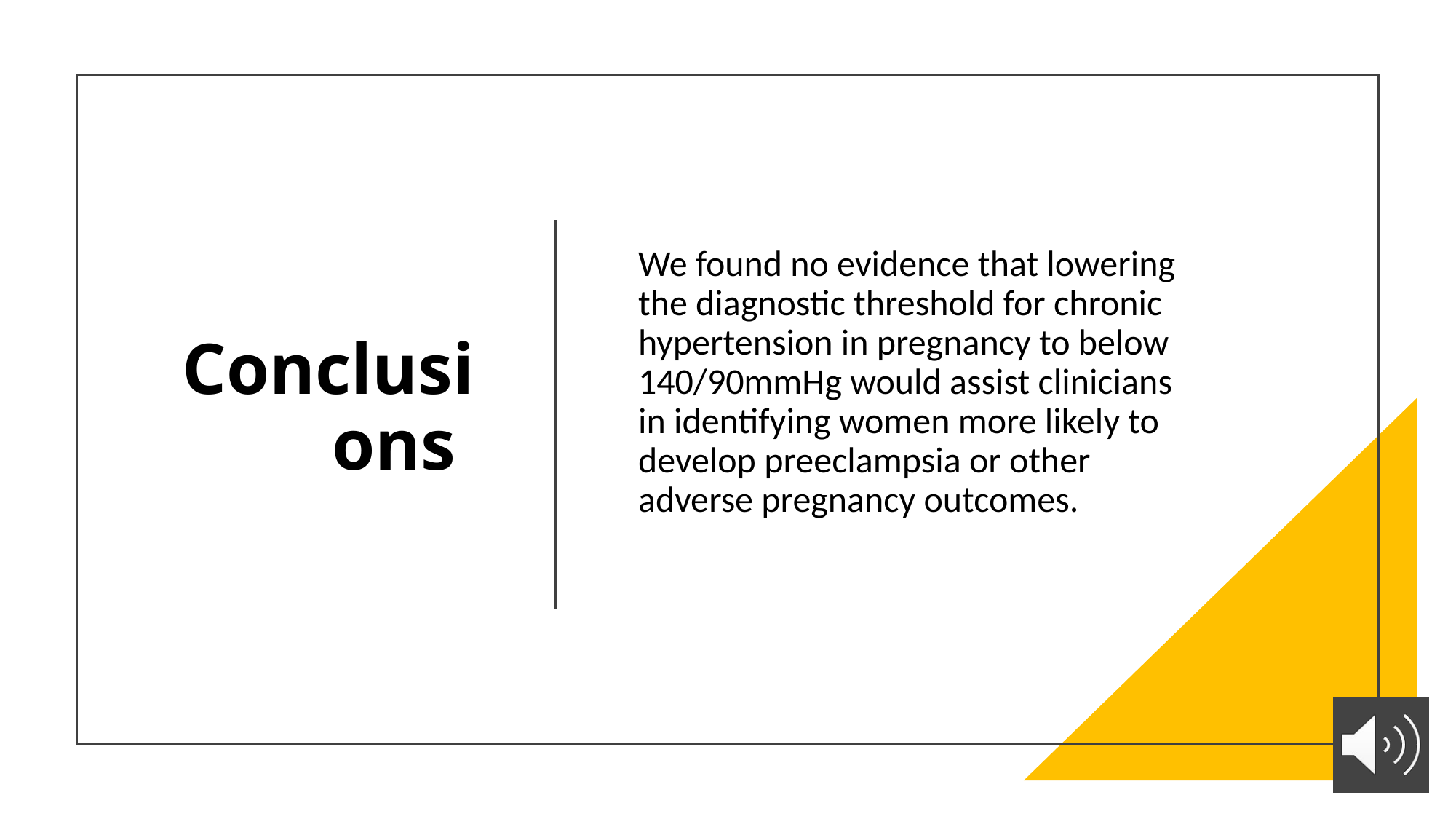

Supplement: AHA BP categories - 10 min [file mmc1.zip › ymob_14772_AHA BP categories - 10 min_mmc1.ppsm]
